# Muscle tissue engineering in fibrous gelatin: implications for meat analogs

**DOI:** 10.1038/s41538-019-0054-8

**Published:** 2019-10-21

**Authors:** Luke A. MacQueen, Charles G. Alver, Christophe O. Chantre, Seungkuk Ahn, Luca Cera, Grant M. Gonzalez, Blakely B. O’Connor, Daniel J. Drennan, Michael M. Peters, Sarah E. Motta, John F. Zimmerman, Kevin Kit Parker

**Affiliations:** 1 Disease Biophysics Group, John A. Paulson School of Engineering and Applied Sciences, Harvard University, Cambridge, MA 02138 USA; 2 Wyss Institute for Biologically Inspired Engineering, Harvard Medical School, Boston, MA 02115 USA; 3 Harvard Stem Cell Institute, Harvard University, Cambridge, MA 02138 USA

**Keywords:** Tissues, Bioinspired materials

## Abstract

Bioprocessing applications that derive meat products from animal cell cultures require food-safe culture substrates that support volumetric expansion and maturation of adherent muscle cells. Here we demonstrate scalable production of microfibrous gelatin that supports cultured adherent muscle cells derived from cow and rabbit. As gelatin is a natural component of meat, resulting from collagen denaturation during processing and cooking, our extruded gelatin microfibers recapitulated structural and biochemical features of natural muscle tissues. Using immersion rotary jet spinning, a dry-jet wet-spinning process, we produced gelatin fibers at high rates (~ 100 g/h, dry weight) and, depending on process conditions, we tuned fiber diameters between ~ 1.3 ± 0.1 μm (mean ± SEM) and 8.7 ± 1.4 μm (mean ± SEM), which are comparable to natural collagen fibers. To inhibit fiber degradation during cell culture, we crosslinked them either chemically or by co-spinning gelatin with a microbial crosslinking enzyme. To produce meat analogs, we cultured bovine aortic smooth muscle cells and rabbit skeletal muscle myoblasts in gelatin fiber scaffolds, then used immunohistochemical staining to verify that both cell types attached to gelatin fibers and proliferated in scaffold volumes. Short-length gelatin fibers promoted cell aggregation, whereas long fibers promoted aligned muscle tissue formation. Histology, scanning electron microscopy, and mechanical testing demonstrated that cultured muscle lacked the mature contractile architecture observed in natural muscle but recapitulated some of the structural and mechanical features measured in meat products.

## Introduction

A key factor limiting the feasibility of bioreactor cultured meat products^1–3^ is an incomplete strategy for adherent cell culture using food-safe processes. This is important, because meat consists of muscle, fat, and connective tissues proportioned according to tissue source,^4^ each containing a diverse array of nutrients produced by constituent cells. Cell types composing meat can be cultured in vitro^5,6^ but production scale-up is limited by the anchorage dependence of these cells, which require attachment to culture substrates for survival, proliferation, and maturation.^7^ This requirement is especially stringent for muscle maturation, where alignment of densely packed muscle fibers is observed.^8–10^ For this reason, controlling cell phenotypes in volumetric cultures is a key challenge for adherent cell bioprocessing,^7,11^ including emerging strategies for meat production.^12–15^ As the design of culture substrates for adherent cell scale-up in food production should also account for emerging food bioprocessing regulatory standards,^16^ we reasoned that fibrous gelatin could fulfill these requirements by recapitulating structural and biochemical features of natural meat tissues.

Natural tissues contain extra-cellular matrix (ECM) protein scaffolds that support cell anchorage and tissue assembly via integrin and other binding sites,^17^ with collagen being the most abundant ECM protein in skeletal muscle, accounting for ~ 1–10% of the muscle mass dry weight.^18^ Collagen and collagen-derived gelatins are used in food and pharmaceutical industries due to their biocompatibility, biodegradability, and weak antigenicity.^19,20^ They are also used to improve cell adhesion to microcarriers in suspension,^11^ and microcarrier bead-to-bead transfer of bovine myoblasts^13^ suggests that these cells can be expanded in volumetric microcarrier-based suspensions. However, these microcarriers do not recapitulate the fibrous architecture of natural muscle and generally require post-culture separation of cells from substrates, complicating culture and harvesting processes. Fibrillar architectures can be recapitulated using fibrous gelatin,^21–26^ but low production rates of electrospinning^21,23–25^ or phase separation^22,26^ limit their scalability for food production. To overcome these limitations and significantly increase fiber production rates, our group developed a suite of fiber production systems^27–29^ that include immersion rotary jet spinning (iRJS),^28^ a dry-jet wet-spinning system. A single laboratory-scale iRJS with a top-loading spin reservoir produces fibers at two to four orders of magnitude higher rates than comparable electrospinning systems^21,23–25^ and on the same order of magnitude as the highest throughput commercial electrospinning systems.^30^ In addition, because fiber production by iRJS does not depend on solution conductivity or electrically grounded collectors, the range of biomaterials that can be produced using food-safe solutions and solvents is greatly expanded. We therefore hypothesized that gelatin fibers produced at high rates by iRJS would support muscle tissue engineering in edible scaffolds at scales required to produce meat analogs.

To verify that gelatin fibers produced by iRJS support muscle tissue engineering, we spun microfibrous gelatin scaffolds and seeded them with bovine aortic smooth muscle cells (BAOSMCs) and rabbit skeletal myoblast cells (RbSkMC). We spun either pure gelatin or gelatin mixed with a microbial crosslinker into precipitation baths containing ethanol:water mixtures. The gelatin we used was porcine, produced from mild acid treatment, with a bloom value estimated to be ~300 (Sigma G2500) and the food-safe crosslinker was a microbial transglutaminase (ActivaT1 mTG). Gelatin solution jets entering the precipitation bath were rapidly dehydrated by the bath ethanol, forming solid fibers that were optionally crosslinked by chemical or enzymatic methods. We measured viscoelastic properties of gelatin solutions with and without microbial crosslinking by rheometry, fiber chemical composition by Fourier transform infrared spectroscopy (FT-IR), and fiber structure by scanning electron microscopy (SEM). To produce meat analogs, we cultured BAOSMC and RbSkMC in the scaffolds and verified cell attachment by immunohistochemical staining. Short-length fibers promoted cell aggregation, whereas long fibers promoted aligned tissue formation. Microstructural analysis by histology and SEM revealed that cultured tissues showed similar collagen or collagen-like protein expression to meat products but lacked the mature contractile architectures observed in natural skeletal muscle. We then used mechanical compression and texture profile analysis (TPA)^31^ to provide proof-of-concept comparisons of cultured meat and natural meat texture using food industry testing methods. Taken together, the ability to control both cell aggregation and alignment in free-floating edible scaffolds, and our ability to scale production sufficiently for TPA analysis, make fibrous gelatin a promising scaffold for engineering meat analogs.

## Results

### Scalable production of gelatin microfibers

**Fig. 1:**
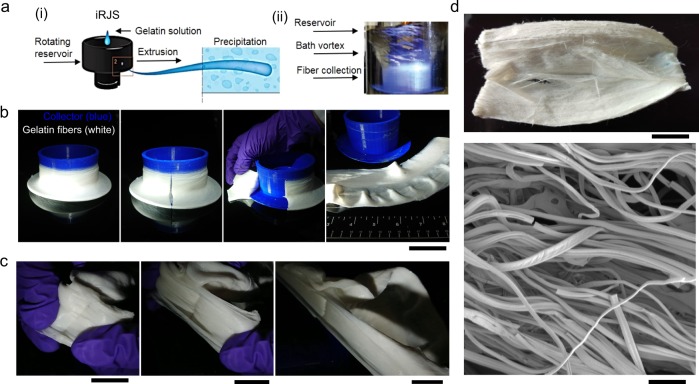
Fibrous gelatin production by immersion rotary jet spinning (iRJS). **a** Schematic (i) and photo (ii) of iRJS fiber production. The schematic shows a precursor solution fed into an open-top rotating reservoir. The solution is extruded through small orifices in the reservoir wall into a precipitation bath where fibers are collected on a rotating cylindrical collector. **b** Removal of gelatin fibers from the iRJS collector following a 10-min production run; scale bar is 10 cm. **c** Peeling fibrous gelatin; scale bar is 1 cm. **d** Freeze-dried fibrous gelatin; scale bar is 1 cm, bottom panel shows scanning electron microscope image; scale bar is 50 μm

In our first set of experiments, we tested the use of iRJS for gelatin fiber production. Using a laboratory-scale iRJS, gelatin was extruded through reservoir wall perforations into an ethanol bath (Fig. 1a), where fibers were guided by the bath vortices to a cylindrical collector at a rate of ~2 g/min (Fig. 1b) and a rotating collection process ensured anisotropic fiber alignment (Fig. 1c, Supplementary Movie 1, and Fig. 1d).

**Fig. 2:**
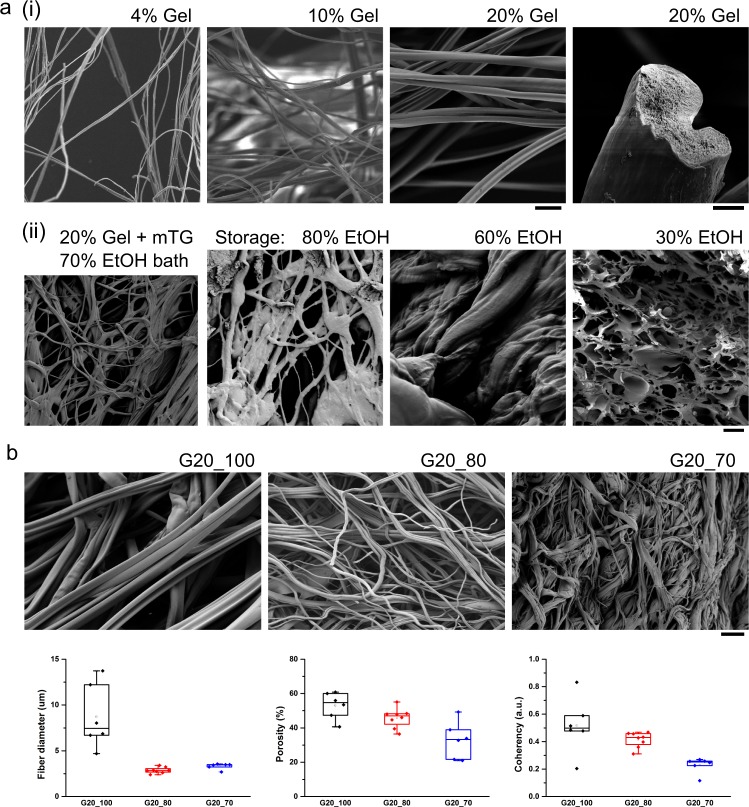
Gelatin fiber morphology and analysis using scanning electron microscopy (SEM). **a** Fibrous gelatin scaffolds (i) produced using three different gelatin concentrations (4%, 10%, and 20% w/w gelatin in DI H_2_O) spun into a pure ethanol (EtOH) precipitation bath; scale bars are 20 μm (left panels) and 2 μm (right panel); (ii) scaffolds produced by co-spinning 20% gelatin and a microbial crosslinking agent into 70:30 EtOH:H_2_O bath, with subsequent storage in EtOH:H_2_O at indicated concentrations (EtOH:H_2_O, from left to right: 100:0, 80:20, 60:40, 30:70); scale bar is 20 μm. **b** Gelatin fibers produced using 20% gelatin spun into EtOH:H_2_O precipitation bath at indicated concentrations (EtOH:H_2_O, from left to right: 100:0, 80:20, 70:30); scale bar is 50 μm. Data plots for fiber diameter, scaffold porosity, and scaffold coherency (alignment) are *N* = 3 productions runs for each bath composition. Coherency depicts alignment ranging from 0 (no alignment) to 1 (perfect alignment). Data are presented as box plots, where lower or upper edges of the box represent 25th or 75th percentiles, the middle bar is the median, and whiskers are 5th or 95th percentiles

Rheological measurements indicated that 20% gelatin solutions behaved like Newtonian fluids with shear rates of 10^–1^ to 10^3^ 1/s and shear stresses of 10^–1^ to 10^3^ Pa (Fig. S1a, b), whereas addition of the mTG crosslinking agent caused a viscous to elastic transition that equilibrated after ~10 min (Supplementary Fig. S1c). These measurements suggested that gelatin fibers could be spun with or without addition of the mTG crosslinker. We therefore expanded the iRJS parameter space to include multiple gelatin concentrations (4%, 10%, and 20%), precipitation bath compositions (ethanol:water ratios between 100:0 and 30:70), and post-spin storage solution compositions (ethanol:water ratios between 100:0 and 30:70), increasing the water concentrations in both the precipitation bath and post-spin storage solutions with the aim of preserving enzymatic crosslinking activity. We obtained gelatin fibers for all tested gelatin concentrations when the precipitation bath was pure ethanol (Fig. 2a.i), with fiber cross-sections (Fig. 2a.i, right panel) showing the morphology expected from diffusion-limited fiber formation consisting of a dense exterior and inner porous region formation.^32^ The presence of amide peaks observed by Fourier transform infrared spectrographs in all samples (Supplementary Fig. S2) confirmed that gelatin peptide bonds were preserved in non-crosslinked gelatin fibers and in fibers that were crosslinked either chemically or enzymatically. Co-spinning gelatin with mTG into 70:30 ethanol:water baths resulted in fibers with moderate inter-fiber fusion (Fig. 2a.ii, left panel) and their storage in ethanol:water mixtures preserved fiber morphology when the storage media water concentration was 20% or less (Fig. 2a.ii, right panels). These experiments demonstrated that we could control gelatin fiber diameter (between ~1 μm and ~10 μm) and scaffold porosity, depending on iRJS parameters, providing experimental control over features that are important for recapitulating natural muscle structure and mechanical properties.

For muscle cell cultures, we aimed to promote cell infiltration through the use of fibrous three-dimensional (3D) scaffolds having minimal inter-fiber fusion. We therefore focused on spinning pure gelatin (20% w/w solution in deionized water) in precipitation baths with high ethanol concentrations, storing the fibers in pure ethanol. We performed three production runs for each of three bath compositions (ethanol:water = 100:0, 80:20, 70:30). Fiber diameter, scaffold porosity, and fiber alignment (coherency) all depended on bath composition (Fig. 2b). In these experiments, fiber diameters ranged between 2.9 ± 0.1 μm (mean ± SEM, ethanol:water = 70:30) and 8.7 ± 1.4 μm (mean ± SEM, ethanol:water = 100:0). Gelatin fiber diameters were in the same order of magnitude as natural collagen fibers^33^ and closely resembled decellularized mammalian muscle tissue (Supplementary Fig. S3). Taken together, this suggested that gelatin fibers produced using iRJS can serve as good host scaffolds for tissue-cultured meat analogs.

### Muscle cell culture and tissue engineering

**Fig. 3:**
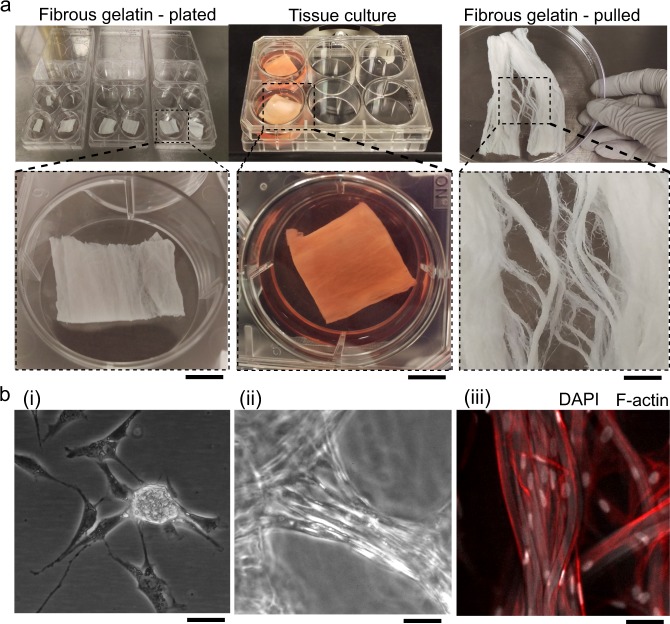
Fibrous gelatin and its preparation for tissue culture. **a** Microfibrous gelatin produced by immersion rotary jet spinning and cut into samples with ~1.5 mm thickness and 6 cm^2^ area were plated individually, seeded with cells and cultured with manufacturer-supplied cell culture media. Scaffolds had multiscale fibrous architectures; scale bars are 1 cm. **b** Rabbit skeletal muscle myoblast cell (RbSkMC) culture and tissue formation depended on gelatin fiber length and crosslinking conditions. We observed (i) spherical aggregates promoted by short-length (~20 μm) fibers, (ii) structurally weak slurry-like tissues resulting from RbSkMC culture in gelatin fibers that were partially crosslinked enzymatically, or (iii) structurally stable tissues resulting from RbSkMC cultured in chemically crosslinked gelatin fibers; cell nuclei are white (DAPI) and the cytoskeleton (F-actin) is red; scale bars are 20 μm

Our cell culture experiments aimed to achieve the following three objectives: (i) verify muscle cell attachment to individual gelatin fibers, (ii) estimate cell density in diffusion-limited thickness (~0.2 mm), enzymatically crosslinked gelatin fiber scaffold volumes, and (iii) achieve long-term culture (21–28 days) in 3D chemically crosslinked scaffolds. For cell adhesion studies, short-length (~10–200 μm) gelatin fibers were transferred by micropipette to glass coverslips. For culture in 3D scaffolds, sections of freeze-dried gelatin fiber scaffolds with area ~4cm^2^ and thickness ~1.5 mm were cultured in multiwell plates (Fig. 3).

First, we cultured BAOSMC and RbSkMC in sparsely distributed short-length (~10–200 μm) gelatin fibers to clarify cell adhesion imaging. Both cell types attached to gelatin fibers, as observed by phase-contrast microscopy (Supplementary Fig. S4a) and immunofluorescent imaging of cytoskeletal (F-actin) and adhesion (vinculin) proteins (Supplementary Fig. S4b, c). Cells formed focal adhesion sites on gelatin fibers (Supplementary Fig. S4b) and cell morphology was directed by the underlying gelatin fibers, aligning on straight fibers (Supplementary Fig. S4b, right panel) or bending on curved fibers (Supplementary Fig. S4c). During our cell attachment experiments, we found that short-length gelatin fibers, with average length ~20 μm, promoted cell aggregation for both BAOSMC (Supplementary Fig. S5) and RbSkMC (Supplementary Figs S6–7 and Supplementary Movie 2), whereas longer fibers promoted aligned tissue formation. Both cell types adhered preferentially to gelatin, despite our use of standard (adherent) tissue culture polystyrene plates (TCPS), which promoted cell monolayer formation in the absence of gelatin fibers (Supplementary Fig. S5). Aggregates assembled within the first week of culture and detached from the substrate by Day 14 (Supplementary Figs S6a), enabling aggregate aspiration by pipette and transfer to fresh plates, confirming the presence of viable proliferative cells in transferred aggregates (Supplementary Figs S6a and S7). Collectively, this indicated that short fibers can be used to generate suspended aggregates, whereas long fibers can be used to control cell morphology in engineered muscle tissues (Fig. 3).

**Fig. 4:**
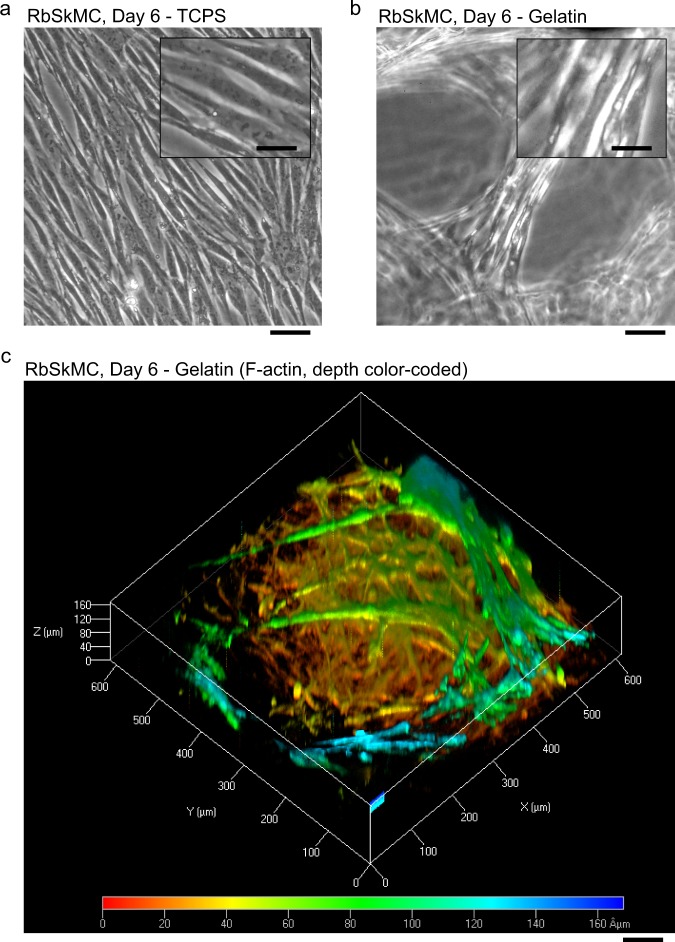
Rabbit skeletal muscle myoblast cells (RbSkMC) cultured in gelatin fiber scaffolds produced by co-spinning gelatin with microbial transglutaminase (mTG). Fibers with average length ~2 cm were distributed on glass microslides (4 cm^2^ area) forming a ~0.2 mm-thick scaffold. **a** RbSkMC monolayers on tissue-culture polystyrene (TCPS) surfaces; scale bar is 100 μm (inset is 30 μm). **b** RbSkMC cultured in partially crosslinked fibrous gelatin-mTG scaffolds; scale bar is 100 μm (inset is 30 μm). **c** 3D reconstruction of cytoskeletal actin filaments (F-actin) in RbSkMC cultured in partially crosslinked gelatin-mTG scaffolds. F-actin stains are color-coded for depth: Red (min = 0 μm) to Blue (max = 170 μm)

When RbSkMC were cultured in gelatin fibers produced by co-spinning gelatin and mTG, a food-safe crosslinking agent, cell viability was maintained within ~0.2 mm-thick tissues for at least 6 days (Fig. 4). In contrast with RbSkMC monolayers cultured on TCPS (Fig. 4a), RbSkMC cultured in anisotropic-aligned gelatin scaffolds formed 3D tissues (Fig. 4b) with cytoskeletal (F-actin) networks visible throughout the tissue volume (Fig. 4c, where RbSkMC density was ~10^4^ cells/mm^3^ and images are depth color-coded). However, fiber morphology and anisotropic tissue alignment were not maintained throughout the entire scaffold volume during the 6-day culture period, owing to a loss of structural integrity observed visually and by microscopy. Enzymatic crosslinking activity had likely been limited by high ethanol concentrations^34^ used in our iRJS spinning baths and storage solutions. For this reason, our long-term cell culture experiments were performed in chemically crosslinked fibrous gelatin scaffolds, where structural anisotropy was expected to be preserved for all subsequent experiments detailed here.

**Fig. 5:**
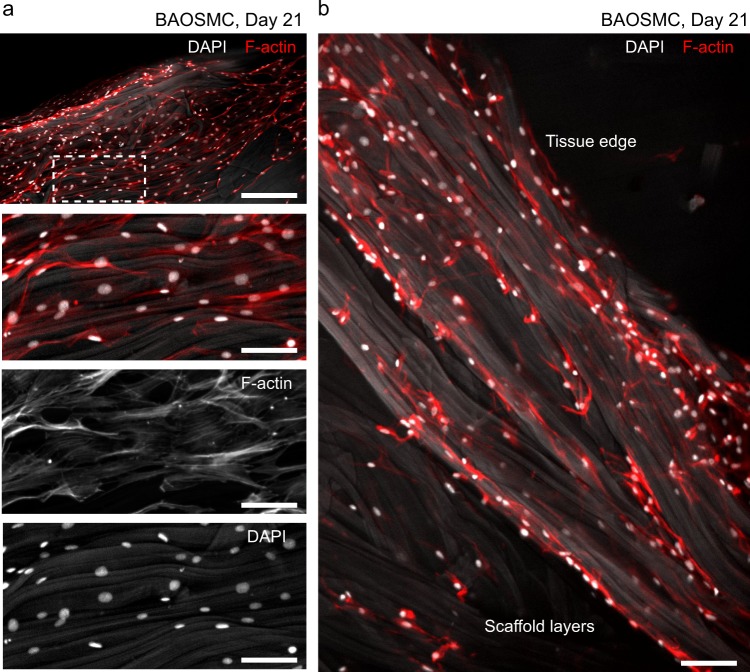
Bovine aortic smooth muscle cells (BAOSMCs) cultured in fibrous gelatin. **a** Immunofluorescent staining of cell nuclei (DAPI, white) and cytoskeletal actin filaments (F-actin, red) showing cell confluence on the surface of a free-floating fibrous gelatin scaffold. Gelatin fibers show as light gray in the DAPI channel; scale bar in the top panel is 200 μm, bottom three panels are 50 μm. **b** BAOSMCs on and below the scaffold surface, showing cells infiltrating the scaffold volume. Scale bar is 50 μm

**Fig. 6:**
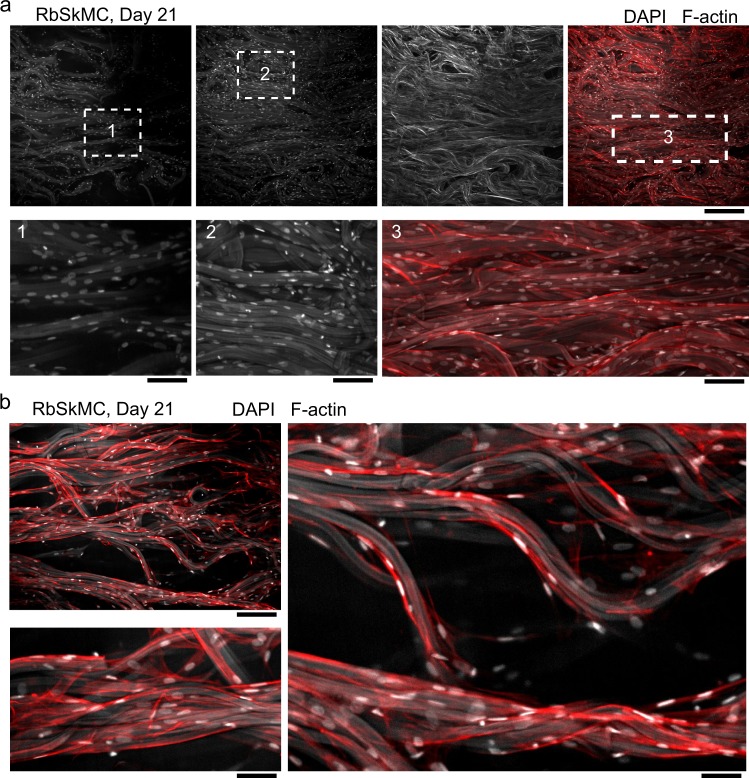
Rabbit skeletal muscle myoblast cells (RbSkMC) cultured in fibrous gelatin. **a** Immunofluorescent staining of cell nuclei (DAPI, white) and cytoskeletal actin filaments (F-actin, red) showing cell confluence on the surface of a free-floating fibrous gelatin scaffold. Surface area for images shown in the top panel are 1 mm^2^. Gelatin fibers show as light gray in the DAPI channel. Magnified views show cell nuclei anisotropy and alignment with the underlying gelatin fibers; scale bars are 200 μm (top row) and 50 μm (bottom row). **b** RbSkMC attachment and alignment in 3D gelatin fiber bundles and threads; scale bars are 200 μm (top left panel), 20 μm (bottom left panel), and 20 μm (right panel)

**Fig. 7:**
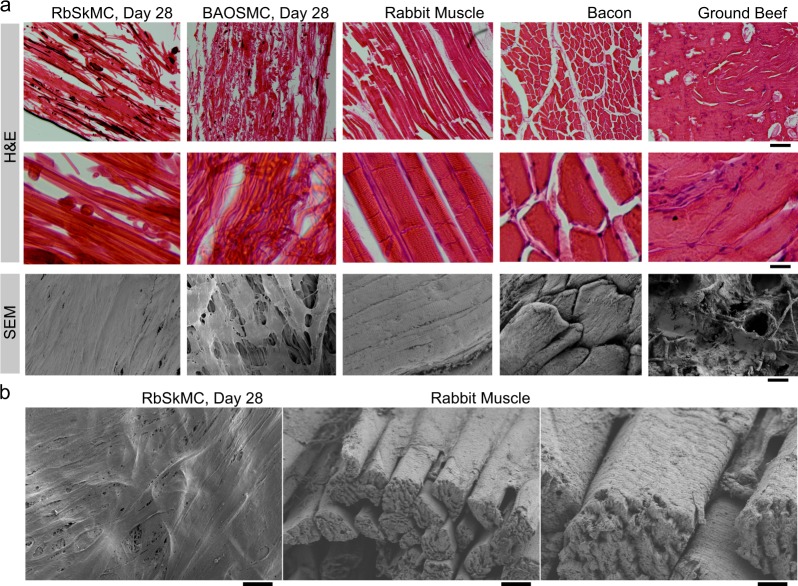
Microstructural comparison of cultured tissues and food products. **a** Hematoxylin and eosin (H&E) stains (top two rows) and scanning electron microscopy (SEM) of rabbit skeletal muscle myoblast (RbSkMC)- and bovine aortic smooth muscle (BAOSMC)-cultured tissues (both Day 28), compared with natural rabbit muscle (freshly isolated gracilis muscle from hind limb), bacon, and ground beef. Scale bars are 200 μm (top row), 50 μm (middle row), and 20 μm (bottom row). **b** SEM images comparing cultured RbSkMC tissue (left panel, scale bar is 10 μm) with skeletal muscle tissue isolated from uncooked rabbit hind limb (right panel, gracilis muscle, scale bar is 10 μm, inset scale bar is 2 μm)

For long-term cell culture experiments, we cultured BAOSMC (Fig. 5) and RbSkMC (Fig. 6) for 21 days in fibrous gelatin scaffolds that were crosslinked with a chemical agent, EDC-NHS (*N*-(3-dimethylaminopropyl)-*N*’-ethylcarbodiimide hydrochloride–*N*-hydroxysuccinimide). Fiber morphology was preserved during culture, as observed by microscopy (Figs 5 and 6) and histological staining (Fig. 7). Cell morphology depended on their culture substrates, with nuclear eccentricity,^35^ 0 ≤ *ϵ* ≤ 1, increasing for BAOSMC when they were cultured on gelatin fibers, compared with two-dimensional (2D) TCPS surfaces: for BAOSMC, *ϵ* = 0.68 ± 0.02 (2D TCPS) and *ϵ* = 0.77 ± 0.02 (3D gelatin fibers). For RbSkMC, *ϵ* = 0.84 ± 0.02 (2D TCPS) and *ϵ* = 0.79 ± 0.03 (3D gelatin fibers). In previous work, we described how substrate mechanics could drive cell nucleus deformation through intracellular force transduction in cultured cardiomyocytes.^35^ Here, nuclear eccentricity was more pronounced for RbSkMC than BAOSMC, in both 2D TCPS and 3D gelatin fibers, as expected for skeletal vs. smooth muscle phenotypes.^36^ Dense tissues were observed in scaffolds having cross-sectional areas >1 cm^2^ for BAOSMC (Supplementary Fig. S8) and RbSkMC (Supplementary Fig. S9 and Supplementary Movie 3). BAOSMC cell density (cells/mm^2^) on 2D TCPS or in 3D gelatin fibers were 624.3 ± 156.5 or 556.9 ± 95.1, and RbSkMC cell density (cells/mm^2^) on 2D TCPS or in 3D gelatin fibers were 357.4 ± 85.1 or 1021.5 ± 226.5, respectively. Successive reduction of F-actin channel intensity (Supplementary Fig. S10) revealed the underlying gelatin fibers and their influence on cell and cell nuclei anisotropy and alignment.

Having verified that BAOSMC and RbSkMC attached to gelatin fibers and formed 3D tissues, we then used histological staining and SEM to compare cultured tissue microstructure with a variety of commercially available food products (Fig. 7). Hematoxylin and eosin (H&E) staining of cultured BAOSMC and RbSkMC tissues cultured for 28 days (Fig. 7, left panels) showed similar collagen or collagen-like protein expression to natural rabbit skeletal muscle, bacon, or ground beef (Fig. 7, left panels). Our cultured samples had aligned fibrous structures but lacked the densely packed striated muscle fibers characteristic of natural rabbit muscle or bacon. This was expected, because (i) RbSkMC are proliferative cells with limited differentiation capacity and therefore limited sarcomere assembly, and (ii) BAOSMC are smooth muscle cells that do not develop skeletal muscle contractile architectures. As a result, our tissue scaffolds were closer in morphology to processed meat products such as “fish balls” or ground beef, which show a less homogeneous tissue distribution (Fig. 7 and Supplementary Fig. 11), likely resulting from disruption of tissue structure and cell nuclei during production. Skeletal muscle isolated from the hind limb gracilis muscle of uncooked rabbit (Fig. 7b, right panel) is an example of skeletal muscle with mature contractile architecture that currently cannot be engineered but provides excellent design criteria for future work.

**Fig. 8:**
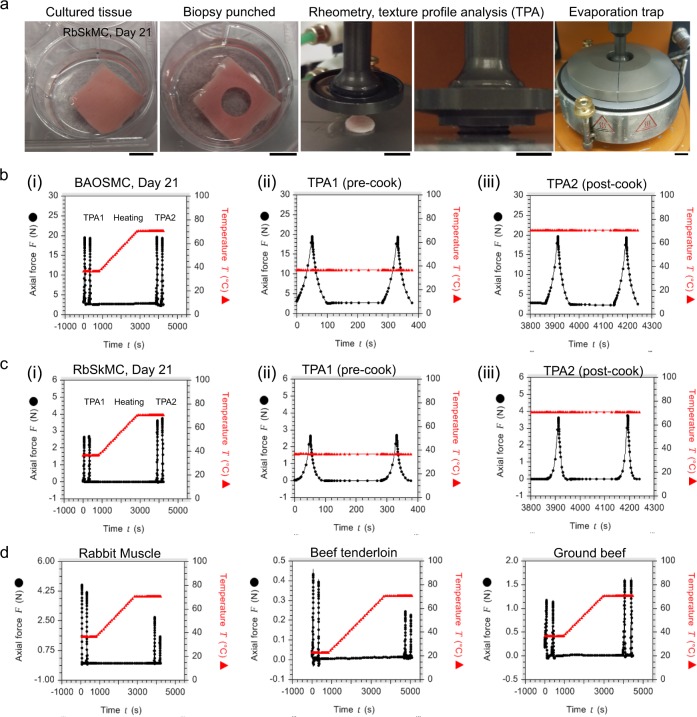
Texture profile analysis (TPA) of cultured tissues and selected food products. **a** Experimental procedure demonstrated using a tissue of rabbit skeletal muscle myoblast cells (RbSkMCs) cultured in fibrous gelatin; samples having 1 cm diameters and ~1.5 mm thickness were obtained by biopsy punch and transferred to the rheometer plate for rheometry and compression tests; scale bars are 1 cm. **b**–**d** Representative force curves obtained by running a TPA cycle at 37 °C (TPA1: two compressions), followed by a temperature ramp to 70 °C, and a second TPA cycle (TPA2: two compressions, cooked). Force (solid black circles) and temperature (solid red triangles) are co-plotted. For BAOSMC **b** and RbSkMC **c** cultured tissues, the pre- and post-cooked TPA curves are shown in panels (ii) and (iii), respectively

Rheological mapping of scaffolds and tissues in frequency and amplitude domains showed little difference within our testing regimes (Supplementary Fig. 12), suggesting that TPA based on sample compression^31^ was more suitable for comparing mechanical properties of cultured tissues with commercially available food products (Fig. 8, Supplementary Figs S13-S14, and Supplementary Table 3). We therefore performed TPA before and after cooking by heating on a rheometer plate (Fig. 8a). Cylindrical samples with 1 cm diameter and 1.5 mm thickness were subjected to dual compressions of 25% strain before and after cooking, producing two pairs of TPA force curves, with each pair separated by a heating regime (Fig. 8b–d). Representative force curves obtained from tissues composed of either BAOSMC (Fig. 8b), RbSkMC (Fig. 8c), and several food products (Fig. 8d) are shown for comparison. TPA curve amplitudes (i.e., “hardness”) decreased after cooking fresh rabbit muscle or beef tenderloin (Fig. 8d, left panels), whereas cultured tissues were unchanged (Fig. 8b; BAOSMC) or increased (Fig. 8c; RbSkMC), similar to the increase observed for ground beef (Fig. 8d, right panel). These differential responses to heating likely resulted from breakdown of the tissue matrix in natural tissues (e.g., collagen solubility), whereas cultured tissues and ground beef showed little change or increased in hardness, because their initial pre-cooked matrix was gelatin-based (cultured tissues) or had been broken and homogenized during production (ground beef). These preliminary results demonstrate that we can scale production of tissue engineered meat analogs for food industry testing methods that use shear or compressive forces to evaluate texture.

## Discussion

The production of meat by cell culture and tissue engineering offers theoretically high resource efficiency, with predicted reductions in land and water use >80% compared with meat produced from livestock.^12,37^ However, further research and development are required to advance cell culture scale-up and production of meat analogs that recapitulate the structure and composition of natural meats.^38^ Here we showed that biomimetic microfibrous gelatin recapitulated key features of meat ECM materials and supported muscle tissue engineering. As a food-safe consumable,^19,20^ fibrous gelatin can form the basis for meat analogs with or without cultured cells and, in the former case, obviates the need for post-culture separation of cells from carrier substrates. Our gelatin fiber-spinning system, iRJS,^28^ begins to scale fiber production closer to industrial demands and iRJS production throughput is expected to increase, given the recent introduction of this technology and its scalable perforated reservoir-based operation. Although we used gelatin exclusively in the present work, iRJS can produce a variety of biomolecular fibers, including polysaccharides and other plant-derived biomolecules^28^, which add nutritional value to edible scaffolds. These methods provide a path forward for meat analog formulation, where texture and biochemistry are first controlled by tailoring the architecture and composition of fibrillar scaffolds, and cell cultures are subsequently used to increasingly recapitulate natural meat.

Our findings confirmed that muscle cells derived from two separate sources, rabbit myoblasts or bovine aortic smooth muscle, both adhered to gelatin fibers and formed aggregates or aligned tissues depending on scaffold properties (fiber length). Short fibers with length less than ~20 μm promoted cell aggregation, whereas long fiber scaffolds with average length greater than ~1 cm promoted alignment. Both tissue types (aggregated or aligned) can be used for cell expansion in volumetric culture systems,^7^ including stacked or rolled sheets, gas-permeable bags, spinner flasks, and bioreactors. Further studies of gelatin fiber aggregation are warranted, given prior successes using aggregates for high-yield suspension culture of adherent cells.^6,39–41^ These include scalable systems with serum-free defined media for embryonic stem cell expansion in aggregate,^39^ pluripotent stem cell expansion in spinner flasks with differentiation to >90% cardiomyocyte purity,^40^ suspension culture of chicken stem cells,^6^ as well as strategies that increase adherent single-cell survival efficiency, growth rates, and yield.^41^ In our work, the main fiber properties that promoted aggregation were as follows: (i) they resisted degradation in culture for sufficient periods to support cell adhesion and aggregation (~3–14 days) and (ii) fiber length was small compared with aggregate diameter (~50–200 μm). For tissue-engineered muscle based on long gelatin fibers, food-safe gelatin crosslinking strategies must be improved in future work to account for variability based on the gelatin source and processing conditions that can lead to variability of crosslinking and cell adhesion. Residual mTG in our enzymatically crosslinked cultured tissues was less than in some commercial food products (Supplementary Tables 1–2), suggesting that mTG crosslinking optimization may result in food-safe cultured tissues. Given that cell attachment to gelatin is efficient for most adherent cell types,^42^ including stem cells,^43^ we expect future work to include stem cell expansion and lineage specification towards phenotypes that constitute meat. Protocols now exist to derive skeletal muscle,^44^ white and brown fat,^45^ and endothelial cells^46^ from human induced pluripotent stem cells (iPSCs), and recent work with livestock iPSC such as chicken^47^ and pig^48^ suggest that iPSC culture protocols are at least partially translatable between species. Livestock stem cell proliferation and differentiation protocols suggest that a variety of cell types found in meat may be expanded in culture, but most mammalian cell bioprocessing applications currently rely on non-adherent cells cultured in suspension. For example, monoclonal antibody production in Chinese Hamster Ovary (CHO) cells constituted most of the ~8.5 metric ton pharmaceutical production in 2010.^49^ Pharmaceutical bioprocessing with CHO cells became widespread following efforts in the mid-1980s to adapt them for growth in suspension^50^ and recent work suggests that altering adhesion-related gene expression can increase the yield of suspension-cultured pluripotent stem cells.^41^

Ultimately, the texture and nutritional output of cultured meat analogs, whether produced in aggregate, anisotropic tissues, or combinations thereof, should be compared with natural meats.^38^ Texture includes a variety of characteristics such as hardness (some authors call it toughness), springiness, and chewiness, with hardness being the most important to the consumer.^51^ To evaluate these characteristics and use them to predict sensory texture, several testing methods have been developed, including TPA^31,51,52^ and Warner–Bratzler shear (WBs) tests.^52,53^ We used TPA to provide proof-of-concept texture analysis because TPA is an established method and because texture parameters assessed by TPA are good predictors of bovine meat sensory texture.^51,52^ We demonstrated that fibrous gelatin scaffolds, cultured muscle tissues, and commercial food products could all be evaluated using both shear and compressive forces (Fig. 8, Supplementary Figs S12–S14 and Supplementary Table 3) using identical sample geometries and testing conditions. These preliminary results suggest that future work should compare TPA, WBs, and other methods familiar to materials science,^54^ as well as effects of sample geometry and heating, to build a thorough description of meat analog texture and mechanics. Recapitulating the nutritional content of natural meats will likely require tissue culture, because nutrients found in meat, including myofibrillar proteins^55^ and diverse bioactive peptides,^56^ are produced by cells that require specific structural and biochemical cues for appropriate lineage specification. Our histology and mechanical testing suggest that although cultured meats based on BAOSMC or RbSkMC show some similarities to natural meats, further work with improved skeletal myoblast differentiation are required to more closely recapitulate the mature contractile architecture observed in natural muscle. Prior work by our laboratory and others demonstrated that recapitulating natural muscle phenotypes in culture required biomimetic culture conditions^36,57–63^ that account for substrate stiffness^57^ and biochemistry,^58^ anisotropic muscle alignment,^36,62^ and chemical factors secreted by supporting cell types.^63^ For these reasons, recapitulating the nutritional profiles of meat will likely require building on bioengineering strategies^64^ that account for multiscale engineering of genetic and epigenetic factors, as well as cell–material and cell–cell interactions that contribute to tissue development. Our results demonstrated that gelatin fibers provide a suitable scaffold to study muscle cell aggregation or formation of 3D aligned tissues. The general nature of cell adhesion to gelatin, and its recognition as a safe edible material, suggest these scaffolds can support a variety of adherent cell types with utility for food bioprocessing. With further research and development, we believe that muscle bioprocessing and tissue engineering will play increasingly important roles in food science.

## Methods

### Materials

We obtained porcine gelatin powder produced from mild acid treatment, with bloom value ~300, from Sigma-Aldrich, St. Louis, MO, USA (Sigma G2500, CAS 9005-70-8, Type 300A). Chemical crosslinking agents obtained from Sigma were EDC (Sigma product #03450) and NHS (Sigma product #130672). Calcium-independent mTG was obtained from Modernist Pantry, Eliot, ME, USA (ActivaT1 mTG) and was used without further purification: this enzyme is supplied as a proprietary formulation with a maltodextrin support (Ajinomoto ActivaTI: 1% enzyme and 99% maltodextrin) and is reported to have a specific activity of 100 U/g.^65^ Unless stated otherwise, gelatin solutions were prepared by dissolving gelatin in deionized water at 50 °C. Prior to fiber spinning, solutions were maintained at 50 °C by storage in a heated water bath.

### Cells

Primary RbSkMCs (Rb150-05, Lot #2430) and BAOSMCs (B354-05, Lot #1190) were obtained from a commercial vendor (Cell Applications, San Diego, CA, USA) and cultured according to the manufacturer’s recommendations.

### Food products

We obtained fresh uncooked rabbit hind limbs, beef tenderloin, and ground beef from a local supplier of fresh meat products. We obtained bacon, prosciutto, cured turkey breast, and processed “fish ball” meat products from local suppliers. All meats were cut into cylindrical samples by circular biopsy punch with 1 cm diameter and ~1.5 mm thickness. To obtain fresh rabbit muscle, we isolated the gracilis muscle from the hind limb and subsequently cut samples as outlined above. These sample geometries were used for rheometry, TPA, and histological sectioning.

### Shear rheology of gelatin solutions

A TA Instruments Discovery Hybrid 3 Rheometer (TA Instruments, New Castle, DE, USA) with a cone plate geometry was used to study 20% (w/w) gelatin solutions and 1:10 ratio solutions of 20% (w/w) gelatin crosslinked with 50% (w/w) mTG solutions. The cone geometry had a 60 mm diameter, 2° angle, and a 400 µm truncation gap. Both the solutions and the rheometer plate were heated to 50 °C before loading and were maintained at 50 °C during testing. Evaporation plates were used to prevent solution loss during experiments. Crosslinked samples were mixed immediately before loading onto the rheometer with minimal conditioning time before starting experiments. Non-crosslinked samples were given 60 s to equilibrate followed by 60 s of pre-shear stress at a rate of 100 s^−1^ prior to collecting data. Solutions were then sampled for 1 h at a 10% strain rate with points recorded every 6 s. Storage and loss moduli, and other rheological parameters, were derived from the data using manufacturer-supplied software (Trios software v4.5.0.42498, TA Instruments, New Castle, DE, USA).

### Immersion rotary jet spinning

The iRJS extrudes precursor solutions into a precipitation bath through perforations in the wall of a rotating reservoir. For gelatin fiber spinning, we built custom-machined stainless steel open-top reservoirs with volume = 5 mL and dual 0.5 mm extrusion orifices in the reservoir walls. For fiber production, reservoirs were fitted to high-speed motors and gelatin was extruded from the rotating reservoir walls at a fixed rotation rate of 15 kRPM. Gelatin solutions were all prepared in deionized water and maintained at 50 °C in a water bath prior to spinning. They were then transferred to 50 mL syringe tubes and fed by controlled air pressure (10 kPa applied pressure) to the spinning iRJS reservoir at a rate of 10 mL/min for a total of 5 min per production run. In preliminary experiments, we verified gelatin fiber production using three different concentrations (4%, 10%, and 20% w/w in deionized water) in pure ethanol baths. We then varied the bath composition (ethanol:water = 100:0, 80:20, 70:30, 60:40, and 30:70) and observations by optical microscopy revealed that bath water concentrations higher than 30% led to fiber fusion and partial scaffold dissolution in the precipitation bath. We therefore spun replicate samples for three fiber-producing bath conditions (ethanol:water = 100:0, 80:20, 70:30), using 20% gelatin and a constant iRJS reservoir rotation rate of 15kRPM. For these experiments using 20% w/w gelatin solutions, three production runs were conducted for each of the three iRJS bath compositions (ethanol:water = 100:0, 80:20, 70:30). The fibrous gelatin production rate using 20% w/w gelatin solutions was ~100 g/h dry weight. For fiber collection, a circulating precipitation bath vortex was maintained during spinning using a rotating collector fixture. The spinning reservoir was lowered into the center of the bath vortex and solution was extruded through the vortex air gap into the circulating bath. Vortex circulation directed fibers to the central rotating collector, where anisotropic fiber scaffolds accumulate by spooling. Unless stated otherwise, gelatin scaffolds were removed from the collector and stored in ethanol:water storage solutions overnight. The scaffolds were then either stored in pure ethanol or crosslinked, washed, freeze-dried, and stored at −20 °C.

### Gelatin fiber crosslinking

Fibers were either crosslinked enzymatically by co-spinning gelatin with mTG or chemically crosslinked using EDC-NHS. Unless stated otherwise, chemical crosslinking of gelatin fibers was done using EDC (479 mg/50 mL) and NHS (115 mg/50 mL) in pure ethanol. Chemical crosslinking was performed for 24 h, to ensure complete crosslinking of the gelatin fibers. For enzymatic crosslinking during fiber spinning, we prepared separate 20% w/w gelatin solutions and 50% w/w mTG solutions, both in deionized water maintained at 50 °C. Immediately prior to spinning, gelatin and mTG solutions were mixed at a ratio of 2:1. Based on rheological measurements, the combined gelatin:mTG solutions continue to flow for ~10–20 min after mixing.

### Quantification of residual mTG

Residual mTG was quantified in gelatin fiber scaffolds before and after cell culture. Briefly, an enzyme-linked immunosorbent assay (ELISA) was used to detect mTG from *Streptomyces mobaraensis* (Zedira, Art# E021). Gelatin fiber scaffolds used in cell culture were centrifuged at 200 × *g* in 5 mL of culture media and the pellet was resuspended at a 1:5 dilution using the sample buffer provided by the manufacturer. Lyophilized gelatin fibers were hydrated in culture media, centrifuged at 200 × *g*, and resuspended at a 1:5 dilution using provided sample buffer. Resuspended samples were homogenized and centrifuged at 10,000 × *g* for 5 min. Supernatants were further diluted at a ratio of 1:10 or 1:100, and analyzed using the mTG ELISA assay according to the manufacturer’s protocol. The concentration of mTG in each supernatant was calculated using a standard curve generated by a nonlinear regression of a four-parameter function.

### Gelatin fiber fractionation

To produce short-length gelatin fibers, we placed scaffolds measuring ~ 5 cm × 2 cm × 0.5 cm into a commercial blender containing pure ethanol and blended the scaffolds for 10 min using the “ice crush” setting. We transferred the crushed fibers to 50 mL falcon tubes where they were left to sediment overnight. The top fractions were then transferred by pipette to fresh storage tubes. This fractionation procedure resulted in a range of fiber lengths (~10–200 μm) suitable for dispersion on glass coverslips where cell attachment to individual fibers could be observed clearly by optical microscopy.

### Fourier transform infrared spectroscopy

FT-IR spectra of gelatin powder and dried fiber scaffolds were obtained using attenuated total reflectance-FT-IR (Lumos, Bruker, MA, USA). The samples were scanned over 600–4000 cm^−1^ with 16 scans. For data plotting, commercially available software, OriginPro 8.6 (OriginLab Corporation, MA, USA) was used to normalize the original spectra from 0 to 1.

### Scanning electron microscopy

The fibers were prepared on SEM stubs and sputter-coated with Pt/Pd (Denton Vacuum, NJ, USA) with a thickness of 5 nm. Field-emission SEM (Zeiss) was used to obtain SEM images of the fibers. Gelatin fibers used for SEM measurements were crosslinked chemically by EDC_NHS to ensure dimensional stability.

### Analysis of fiber diameter and alignment

ImageJ software (NIH) with the DiameterJ and OrientationJ plug-ins was used to determine fiber diameter and alignment from the SEM images of the fibers as described in previous studies.^66,67^ Coherency depicts alignment ranging from 0 (no alignment) to 1 (perfect alignment).

### Cell culture

Primary RbSkMC (Rb150-05, Lot #2430, 1st passage) and BAOSMCs (B354-05, Lot #1190, 2nd passage) obtained from a commercial vendor (Cell Applications, San Diego, CA, USA) were cultured according to manufacturer recommendations. Both cell types were thawed and plated in 75 cm^2^ TCPS flasks at a density of ~2.5 × 10^3^ cells/cm^2^ (two flasks per cell vial; 0.5 M cells per vial) where they proliferated for 48 h. We passaged the cells one time by trypsinization and centrifugation, replating them at ~2.5 × 10^3^ cells/cm^2^ into eight flasks (total cell number ~2.0 M cells per original 0.5 M cell vial) where they proliferated to a total volume of ~8.0 M cells. Unless stated otherwise, the resulting cells were seeded at the same density (~2.5 × 10^3^ cells/cm^2^) in gelatin fiber samples contained in six-well plates. Cell counting was done using a hemocytometer. For adhesion studies, cells were seeded on sparse gelatin fibers for up to 6 days. For culture in gelatin scaffolds that were partially crosslinked enzymatically, cells were cultured for up to 6 days. For culture in chemically crosslinked gelatin scaffolds, cells were cultured for up to 28 days in scaffolds (scaffold thickness ~1.5 mm, scaffold area ~5 cm^2^). In all cases, the cell culture media used during the first 6 days of culture was manufacturer-supplied proliferation media, Rabbit Skeletal Muscle Cell Growth Medium Kit (Rb151K) for RbSkMC or Bovine Smooth Muscle Cell Growth Medium Kit (B311K) for BAOSMC, replenished daily. For chemically crosslinked gelatin fiber scaffolds seeded with RbSkMC, differentiation media (Rb151D) was supplied every three days for culture days 7–28.

### Immunohistochemical staining and imaging

Cells were fixed by 4% paraformaldehyde with 0.05 % Triton-X 100 for 10 min. The fixed samples were washed three times by phosphate-buffered saline (PBS; Gibco, Thermo Fisher Scientific, USA). The fixed cells were incubated with a primary antibody (anti-vinculin, Abcam, USA) in PBS for 2 h at room temperature, followed by three times PBS wash (10 min per each). Then, the samples were incubated with 4′,6-diamidino-2-phenylindole dihydrochloride (DAPI; Molecular Probes, Thermo Fisher Scientific, USA), Alexa Fluo^TM^ 647 Phalloidin (Molecular Probes, Thermo Fisher Scientific, USA), and a secondary antibody (rabbit IgG (H + L) conjugated to Alexa Fluor® 488; Invitrogen, Thermo Fisher Scientific, USA) for 1 h at room temperature. After incubation, the samples were washed by PBS three times (10 mins per each) and mounted on glass slides for imaging. The immunostained samples were imaged by using a spinning disk confocal microscope (Olympus ix83, USA) Andor spinning disk). The 3D reconstruction of *z*-stacked images was performed by using Zen software (Zeiss, USA).

### Cell density measurements

Cell density were determined for 2D and 3D culture using NIH’s ImageJ. Briefly, 3D *z*-stacks containing DAPI-stained nuclei were projected onto a single field of view, using a maximal intensity projection. To remove fiber autofluorescence, each field of view was background subtracted using a sliding paraboloid reference frame filter, which was applied evenly across each sample. Images were then converted to binaries using global intensity thresholding. To filter out noise, binary images were then “eroded” and “dilated” to remove isolated single pixels and then 2D water shed segmentation was applied to separate convolved nuclei. Each resulting nucleus was then counted using particle analysis and cell densities were normalized over the total area of the field of view. Each nucleus was also fit with an ellipse to measure the semi-major, *a*, and semi-minor axis, *b*, of the ellipse, and to determine nuclear eccentricity, *ϵ*, which is given by the following equation:

$${\it{ \in }} = \sqrt {1 - \frac{{b^2}}{{a^2}}}$$

### Histology

Tissue morphology and structure were assessed using H&E staining. Briefly, after fixation in 4% formalin and paraffin embedding, 20 µm-thick slices were cut longitudinally from cultured samples, prosciutto, bacon, turkey, fish balls, rabbit, and ground beef with a sliding microtome at room temperature. Slices were then stained with H&E, imaged with a Leica CM1950 microscope, and processed with Cellsense software.

### Shear rheology and TPA of food and cultured tissues

TPA was performed using a Discovery Hybrid 3 Rheometer (TA Instruments) with a 20 mm plate geometry on cultured samples, gelatin fiber scaffolds, and a variety of meat samples. Evaporation plates were used to prevent solution loss during experiments. Tissue-cultured samples were tested on the rheometer after 21 days in culture at 37 °C in 1 cm circular samples (~1.5 mm thick) with the rheometer pre-heated to 37 °C for experiments. Samples were then tested using either a two-cycle compression–relaxation TPA procedure or the same TPA procedure followed by frequency and amplitude mapping on raw samples, a cooking stage, post-cooked frequency and amplitude mapping, and a final TPA step. Initial loading gaps were determined by sample thickness, which was used to set vertical displacement and displacement rates so that the sample would undergo a 25% compression for 50 s, a 50 s withdrawal, a 180 s relaxation, followed by another compression-withdrawal process. Frequency and amplitude mappings were performed at 37 °C and 71 °C in cultured samples, and commercial meats were tested at 23 °C and 71 °C. Frequency maps were sampled logarithmically from 10^–1^ to 10^1^ Hz at a 1% strain rate and sampled at 10 points per decade. Amplitude mapping was similar from 10^–1^ to 10^1^ strain % at 10 points per decade. Cultured samples and gelatin fibers were compared to determine whether sample mechanics were scaffold dominated or whether the cultured cell types made a significant contribution to the texture properties of the material. Cultured samples were also compared with real meat samples to study what properties the synthetic samples maintained relative to commercial and butchered products. For TPA parameter analysis, we used manufacturer-supplied software (Trios software v4.5.0.42498, TA Instruments, DE, USA) to obtain values of maximum force during compression (hardness, N), the area under each force curve (N.s), and thereby estimate TPA parameter values following previously published methods.^31^

### Statistical analysis

All data are presented as box plots with all data points overlapping. The edges of the box plots were defined as the 25th and 75th percentiles. The middle bar is the median and the whiskers are the 5th and 95th percentiles. One-way analysis of variance with the post hoc Tukey’s test in OriginPro 8.6 software (OriginLab, MA, USA) was used for statistical comparisons. Statistical significance was determined at *p < 0.05.

### Reporting summary

Further information on research design is available in the Nature Research Reporting Summary linked to this article.

## Supplementary information

Supplemental Information

Reporting Summary

Supplementary Movie 1

Supplementary Movie 2

Supplementary Movie 3

## Data Availability

The authors can confirm that all relevant data are included in the paper and/or its Supplementary Information files.

## Supplementary information

**Supplementary information** accompanies the paper on the *npj Science of Food* website (10.1038/s41538-019-0054-8).
