# Supplementary material for: Muscle tissue engineering in fibrous gelatin: implications for meat analogs
Source: NPJ Sci Food. 2019 Oct 21;3:20. doi: 10.1038/s41538-019-0054-8 (PMC6803664; doi:10.1038/s41538-019-0054-8)
Supplement: Supplementary file 1 — Supplemental Information [file 41538_2019_54_MOESM1_ESM.pdf]

SUPPLEMENTAL MATERIAL

Muscle tissue engineering in fibrous gelatin: Implications for meat analogues

Luke A. MacQueen[1,2,3], Charles G. Alver[1,2,3], Christophe O. Chantre[1,2,3], Seungkuk Ahn[1,2,3], Luca Cera[1,2,3], Grant M. Gonzalez[1,2,3], Blakely B. O'Connor[1,2,3], Daniel J. Drennan[1,2,3], Michael M. Peters[1,2,3], Sarah E. Motta[1,2,3], John F. Zimmerman[1,2,3], Kevin Kit Parker[1,2,3]

[1]Disease Biophysics Group, John A. Paulson School of Engineering and Applied Sciences, Harvard University, Cambridge, MA 02138, USA.

[2]Wyss Institute for Biologically Inspired Engineering, Harvard Medical School, Boston, MA 02115, USA.

[3]Harvard Stem Cell Institute, Harvard University, Cambridge, MA 02138 USA

SUPPLEMENATY FIGURES

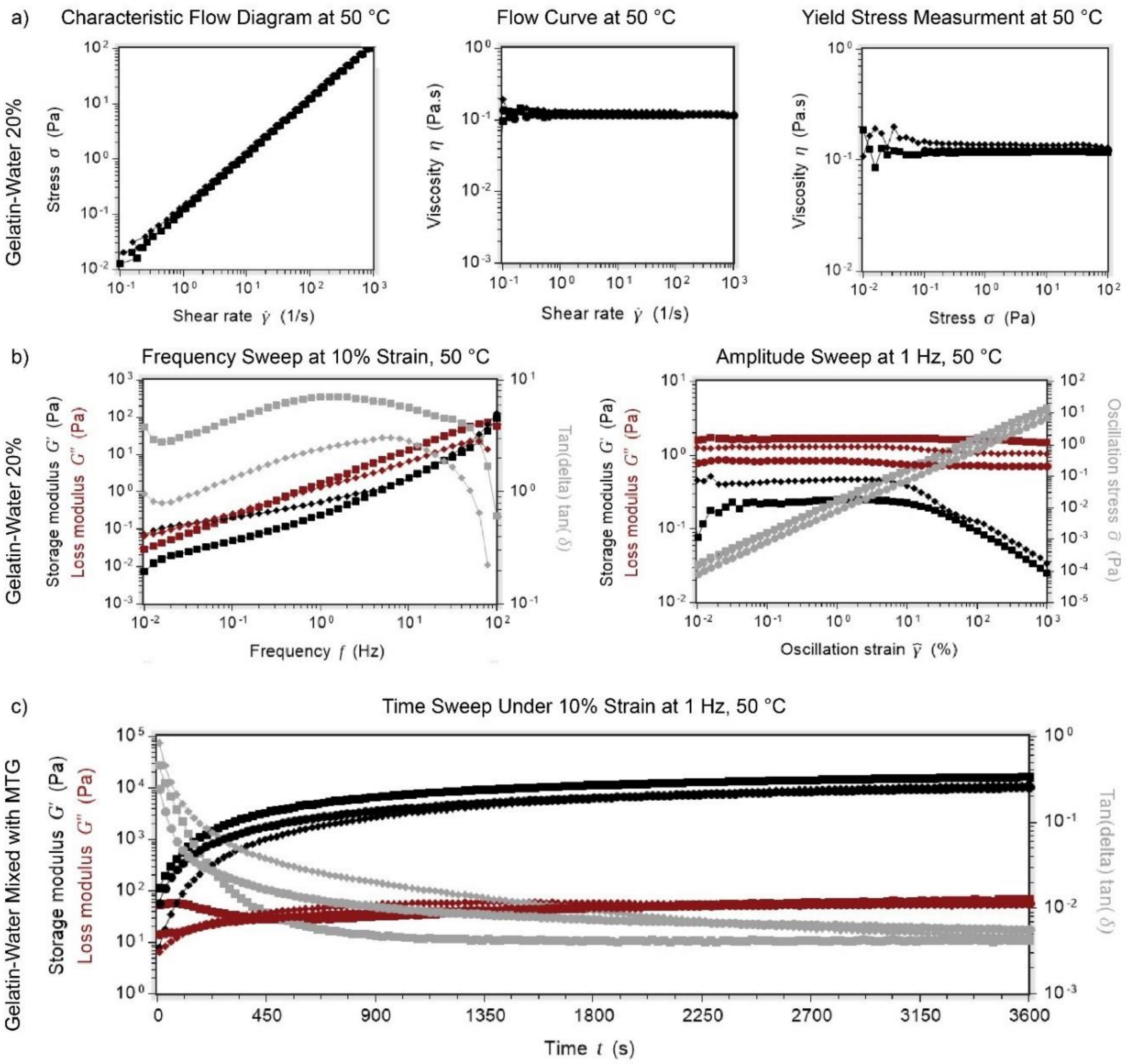

Figure S1 Rheological Mapping of Gelatin Solution a) Characteristic flow diagram, flow curve, and yield stress measurements of gelatin-water 20% (w/w) solutions at 50°C. b) Oscillatory mapping of gelatin-water 20% (w/w) solutions in frequency (constant 10% strain) and strain domains (constant 1 Hz) at 50°C. c) Time sweep of gelatin solutions mixed with MTG crosslinker at 50°C, 1 Hz frequency and under 10% strain. Time sweep begins 30 seconds after initial mixing to due to the time required for mixing and removing loading effects on the sample. Each curve of the same measurement repents independent replicates.

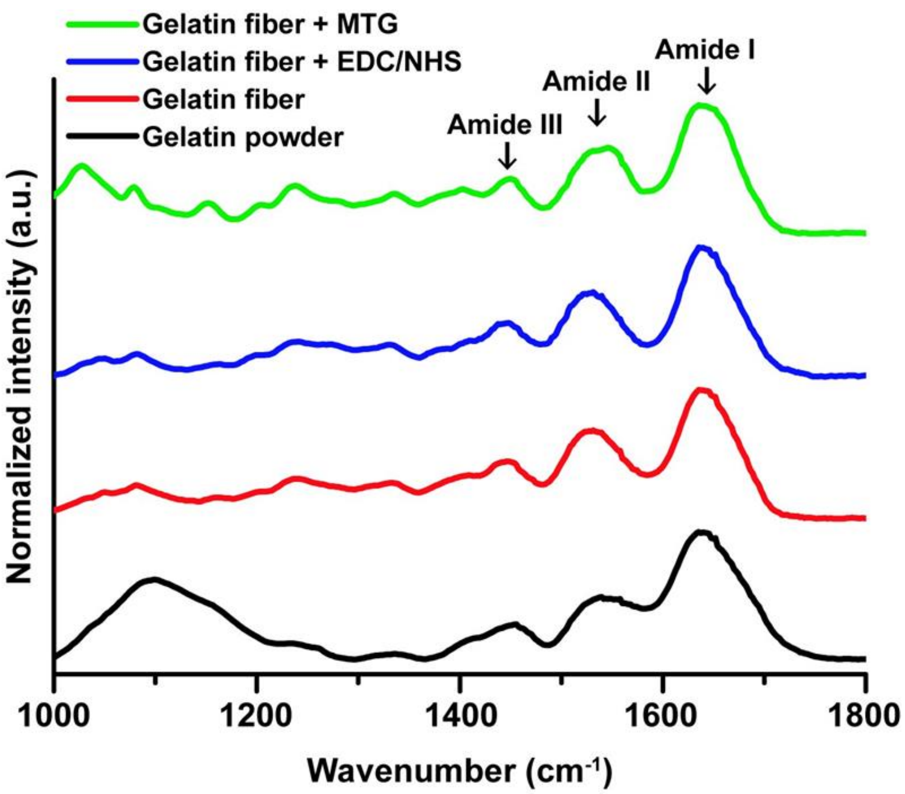

Figure S2 Fourier transform infrared spectroscopy of gelatin powder compared with gelatin fibres. Gelatin powder was porcine type 300A. In all cases, gelatin fibres were spun by immersion rotary jet spinning using a 20 % w/w gelatin precursor solution with gelatin dissolved in DI H_2O at 50 °C. Gelatin fibres crosslinked using microbial transglutaminase (green), EDC-NHS (blue), or non-crosslinked (red) all show amide peaks preserved during the spin and crosslinking processes.

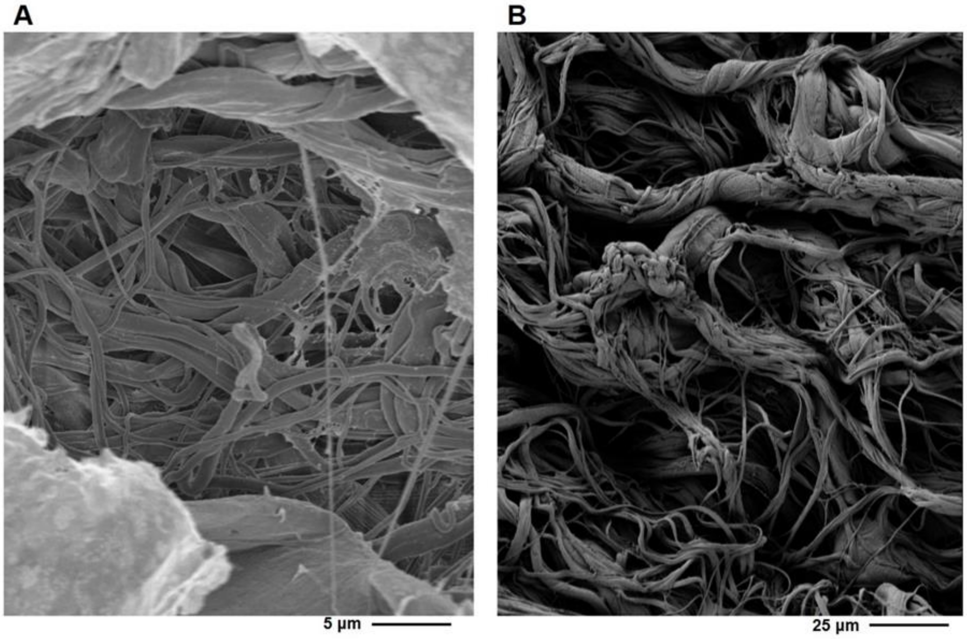

Figure S3 Scanning electron microscopy images of decellularized rat hind limb skeletal muscle (A) and microfibrous gelatin (B). Gelatin was spun by immersion rotary jet spinning, using a 20 % w/w gelatin precursor solution (dissolved in DI H_2O at 50 °C) spun into a precipitation bath with 70:30 ethanol:water composition

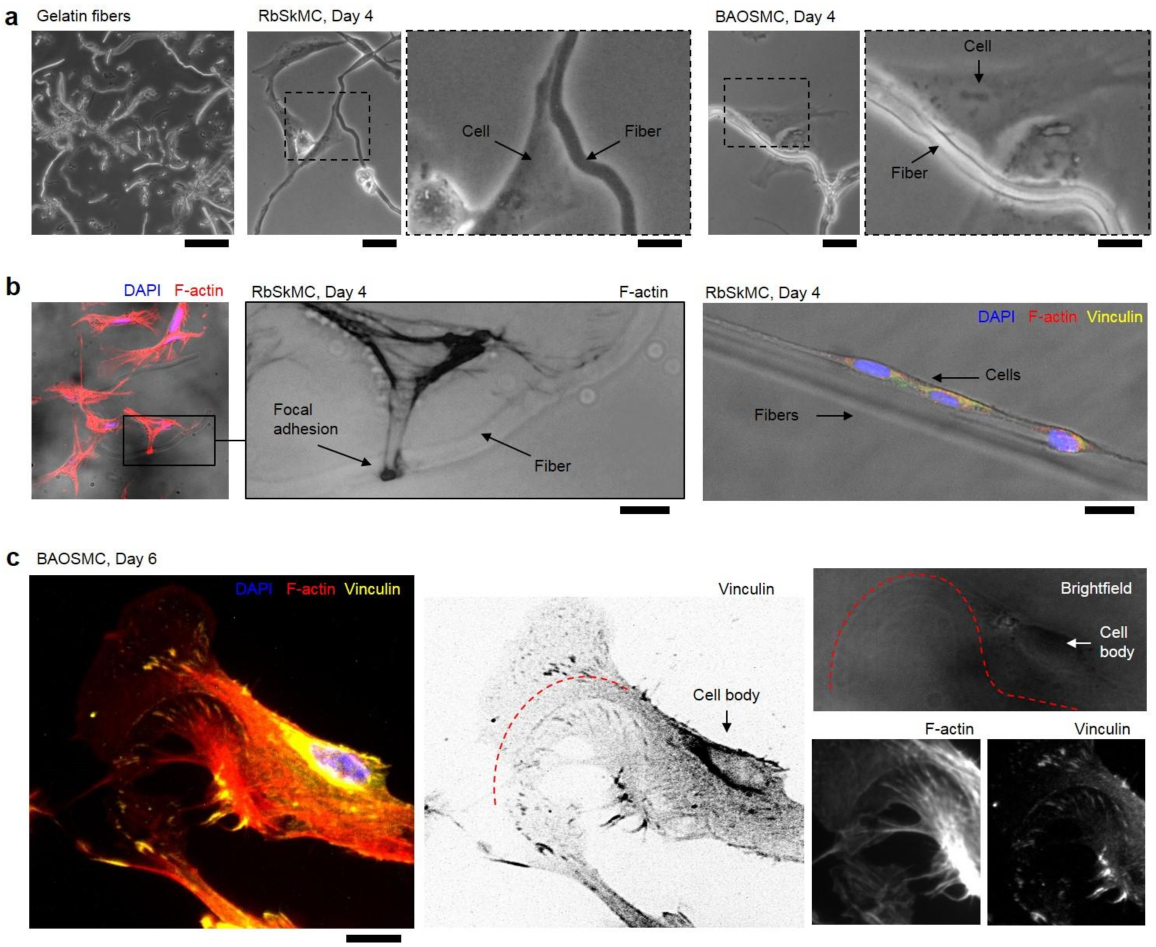

Figure S4 Muscle cell adhesion to gelatin fibers. **a** Brightfield microscopy of sparsely distributed gelatin fibers (left panel), rabbit skeletal muscle myoblast cell (RbSkMC) adhesion to gelatin fibers (middle panels), and bovine aortic smooth muscle cell adhesion to gelatin fibers (right panels); scale bars from left to right are 100 µm, 30 µm, 10 µm, 30 µm, and 10 µm. **b** Immunohistochemical staining of cytoskeletal (F-actin) and adhesion (vinculin) proteins in RbSkMC attached to gelatin fibers. Cell morphology was consistent with attachment to and alignment with underlying gelatin fibers; scale bars from left to right are 10 µm and 20 µm. **c** Immunohistochemical staining of cytoskeletal (F-actin) and adhesion (vinculin) proteins in BAOSMC attached to a curved gelatin fiber: The red dotted line in the middle and top-right panels indicates fiber shape. Cell morphology was consistent with attachment to and alignment with the underlying gelatin fiber; scale bar is 10 µm.

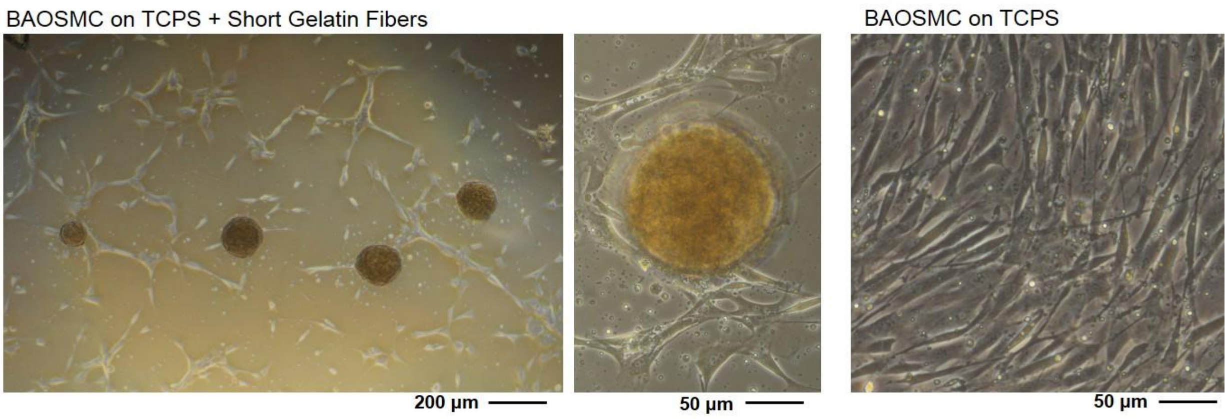

Figure S5 Aggregation of bovine aortic smooth muscle cells (BAOSMC) in short length gelatin fibres (i) compared with BAOSMC cultured on tissue culture polystyrene (ii, TCPS). Aggregates and TCPS samples shown here are Day 4.

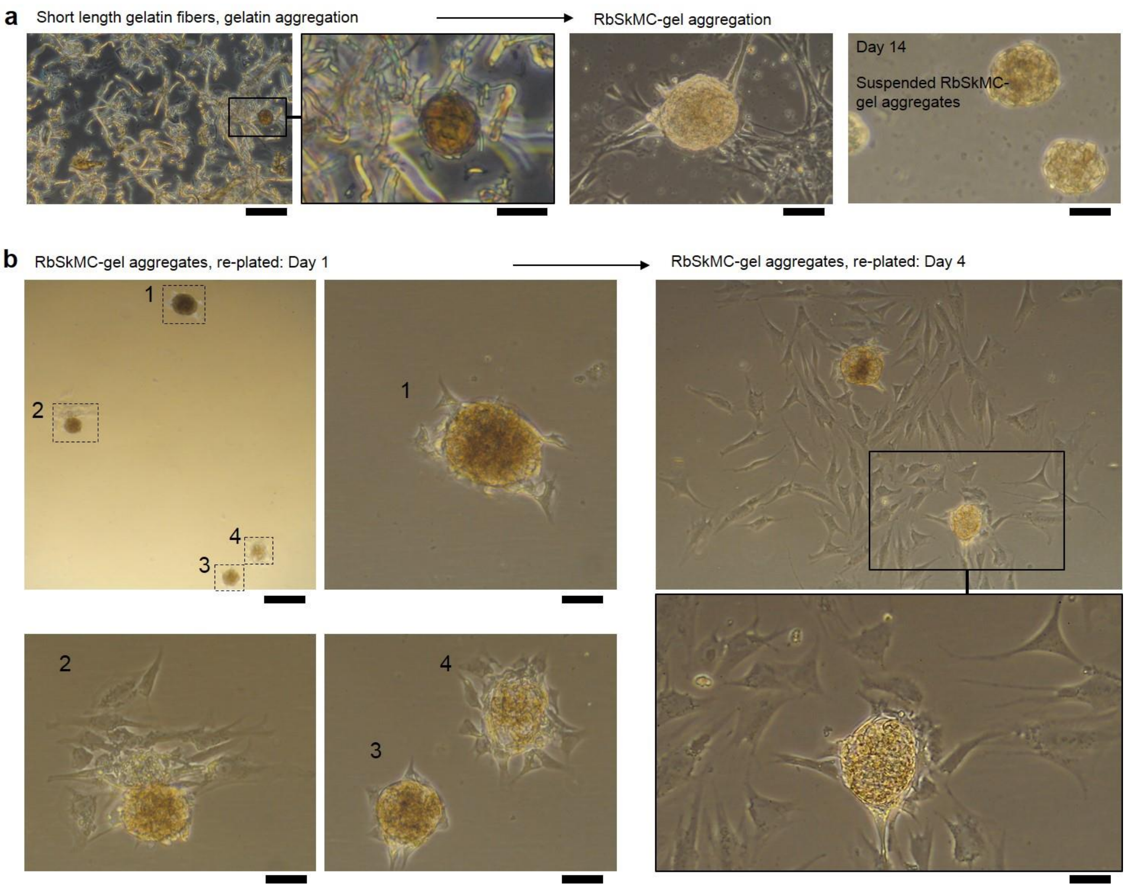

Figure S6 Myoblast aggregates formed in short length (~10-50 µm) gelatin fibers. **a** Brightfield microscopy showing gelatin fibers before cell seeding (left) and after seeding with rabbit skeletal muscle myoblast cells (RbSkMC, right); scale bars from left to right are 200 µm, 50 µm, 50 µm, and 50 µm. Aggregates formed within the first week of culture were suspended (floating) by Day 14 (right most panel). **b** RbSkMC-gelatin aggregates transferred to a fresh flask. RbSkMC cells exiting the aggregate periphery on Day 1 (left panels) proliferated with a doubling time of ~ 1 day (right panels show Day 4); scale bars in the left panel s, top row, from left to right are 200 µm and 50 µm, bottom row are both 50 µm, right panel is 25 µm.

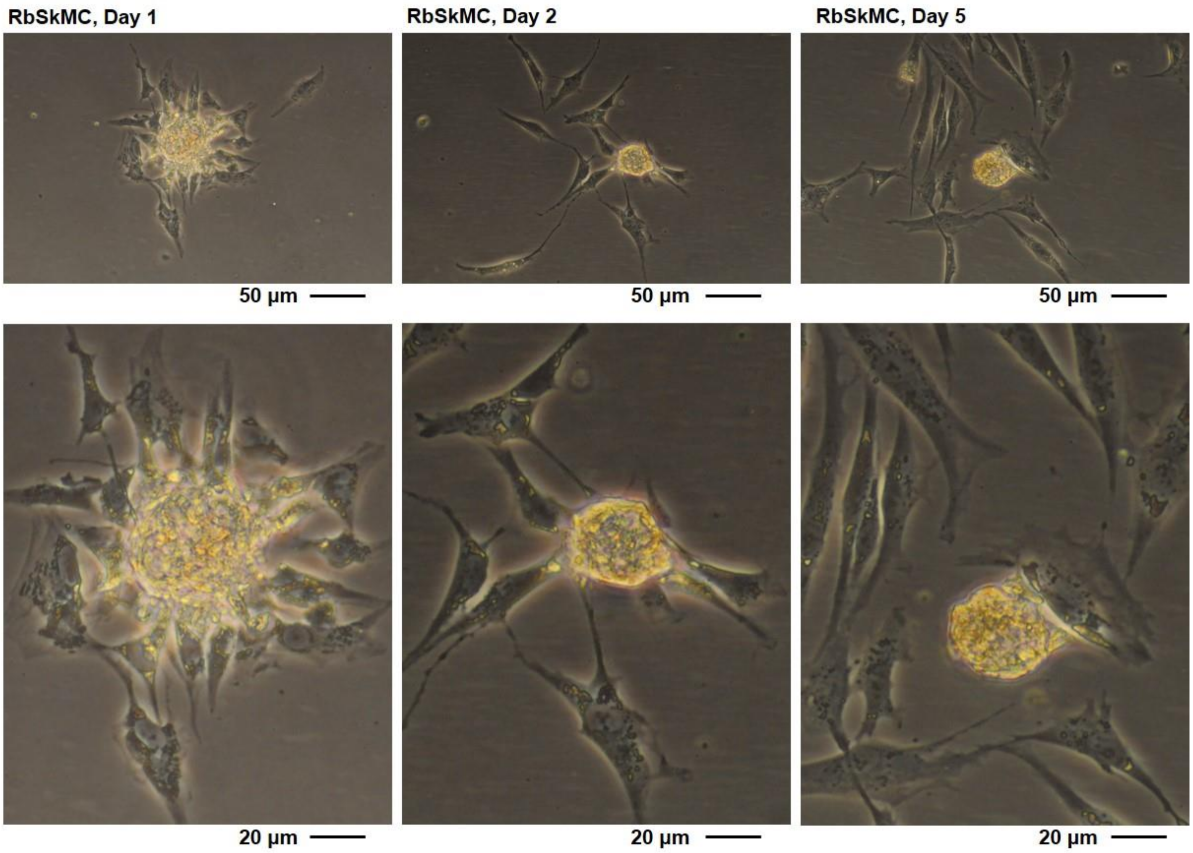

Figure S7 Proliferation of rabbit skeletal muscle myoblast cells (RbSkMC) from RbSkMC:gelatin aggregates. The aggregates were assembled in tissue culture polystyrene plates containing dispersed short length (~20 µm) gelatin fibers. After 14 days in culture, the resulting aggregates were transferred by micropipette to fresh flasks to confirm cell viability and proliferation following transfer. These brightfield microscopy images confirmed RbSkMC transferred as aggregates proliferated at rates comparable to non-aggregated counterparts (~daily doubling).

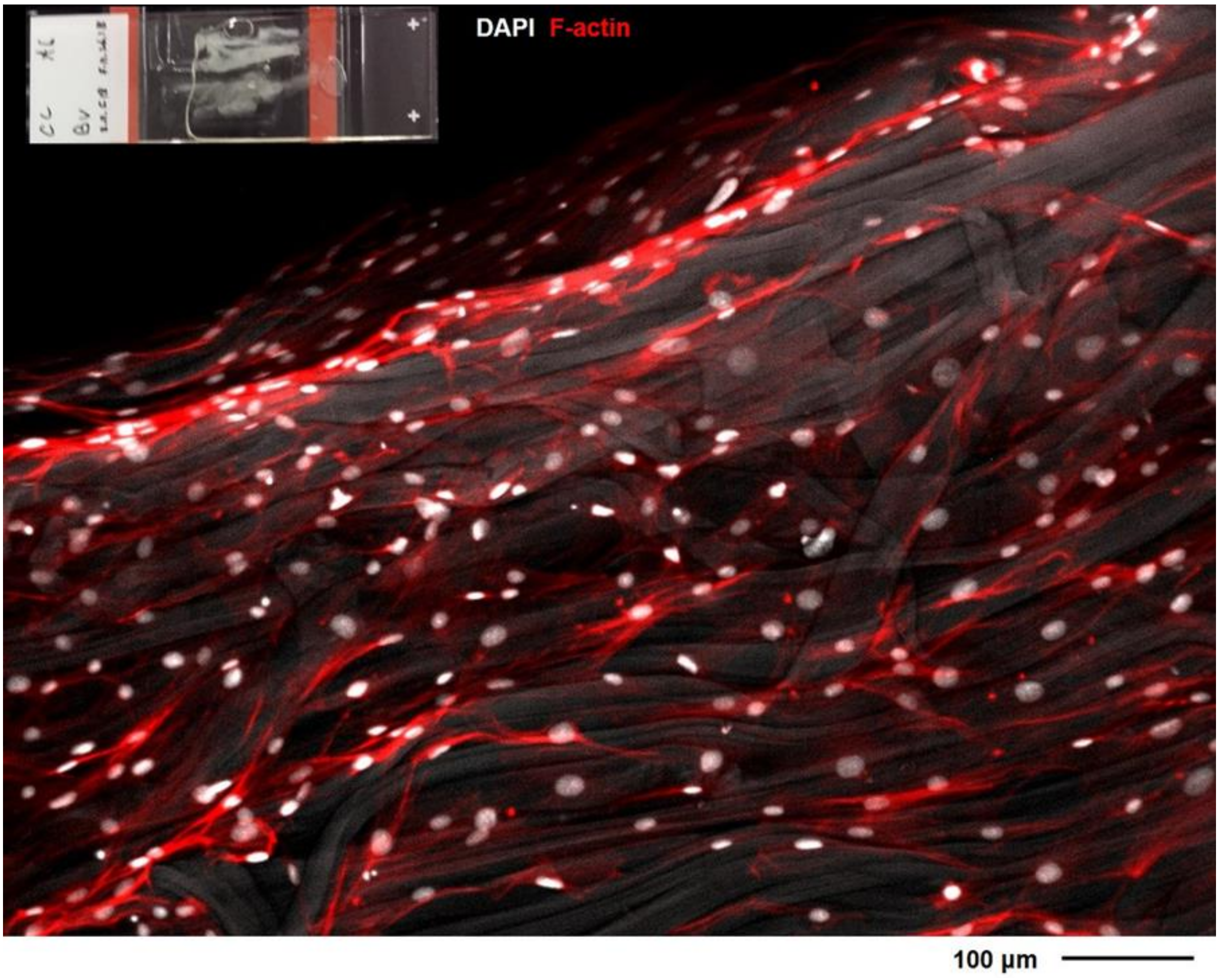

Figure S8 Bovine aortic smooth muscle cell (BAOSMC) tissue formation on a 3D biomimetic fibrous gelatin scaffold. Immunohistochemical staining of filamentous actin (F-actin, Red) and cell nuclei (DAPI, white) confirmed cell confluence and cell alignment, guided by the underlying fibrous gelatin, which is observable as light-grey resulting from gelatin autofluorescence in the DAPI channel. Culture day is 21.

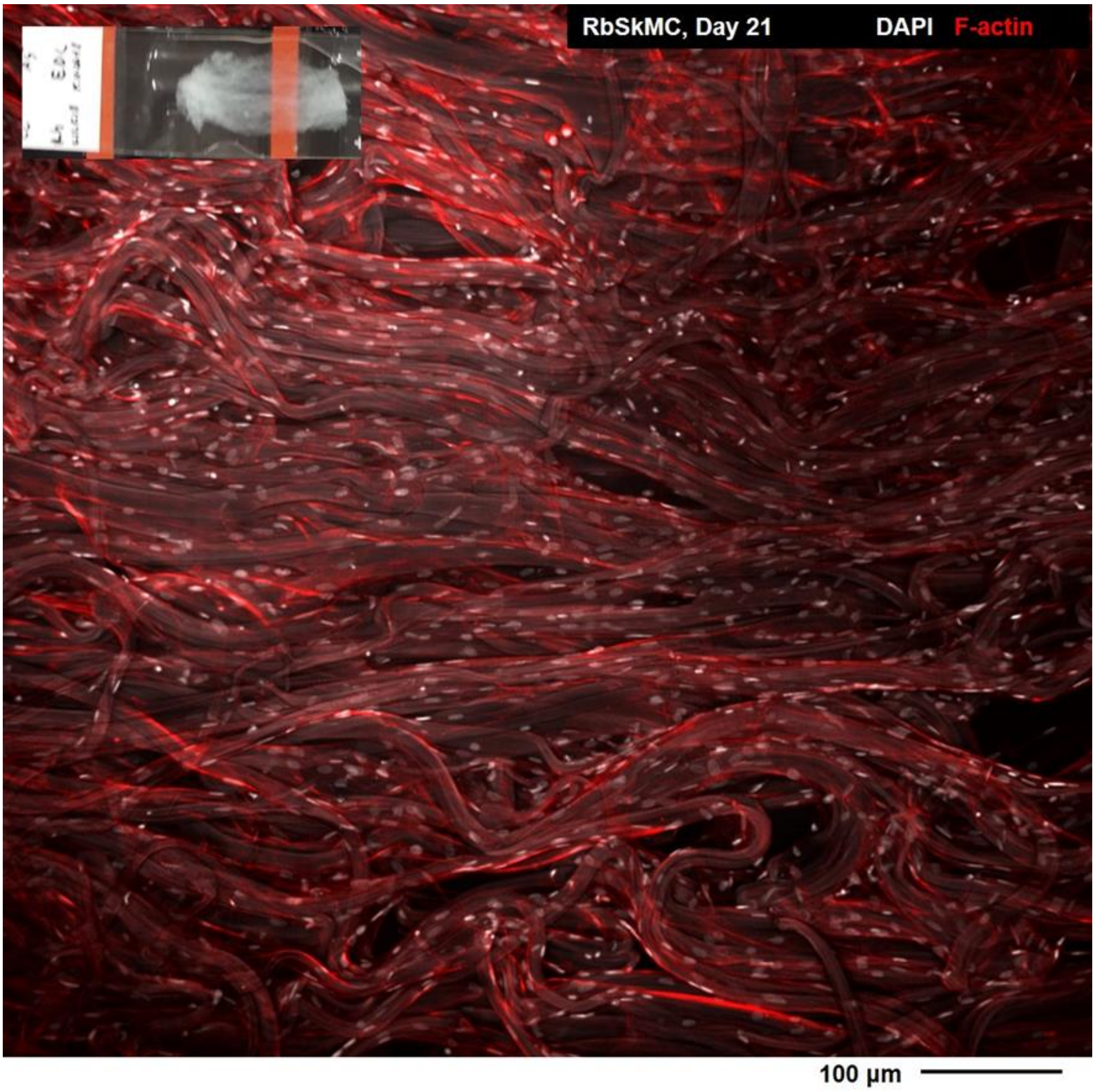

Figure S9 Rabbit skeletal muscle myoblast cell (RbSkMC) tissue formation on a 3D biomimetic fibrous gelatin scaffold. Immunohistochemical staining of filamentous actin (F-actin, Red) and cell nuclei (DAPI, white) confirmed cell confluence and cell alignment, guided by the underlying fibrous gelatin, which is observable as light-grey resulting from gelatin autofluorescence in the DAPI channel. Culture day is 21.

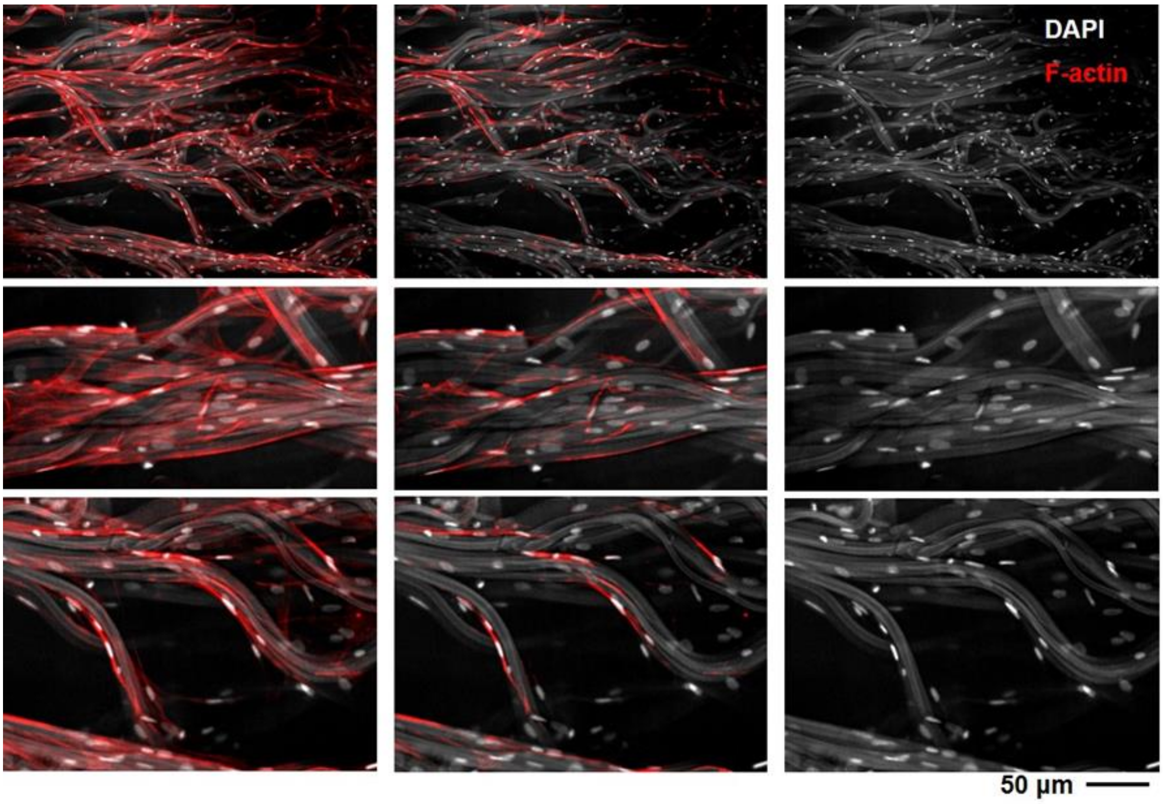

Figure S10 Rabbit skeletal muscle myoblast cell (RbSkMC) tissue formation on a 3D biomimetic fibrous gelatin scaffold. Immunohistochemical staining of filamentous actin (F-actin, Red) and cell nuclei (DAPI, white) confirmed cell confluence and cell alignment, guided by the underlying fibrous gelatin, which is observable as light-grey resulting from gelatin autofluorescence in the DAPI channel. Successive reduction of F-actin channel intensity (from left to right) reveals the underlying gelatin fibres and their influence on cell and cell nuclei anisotropy and alignment. Culture day is 21.

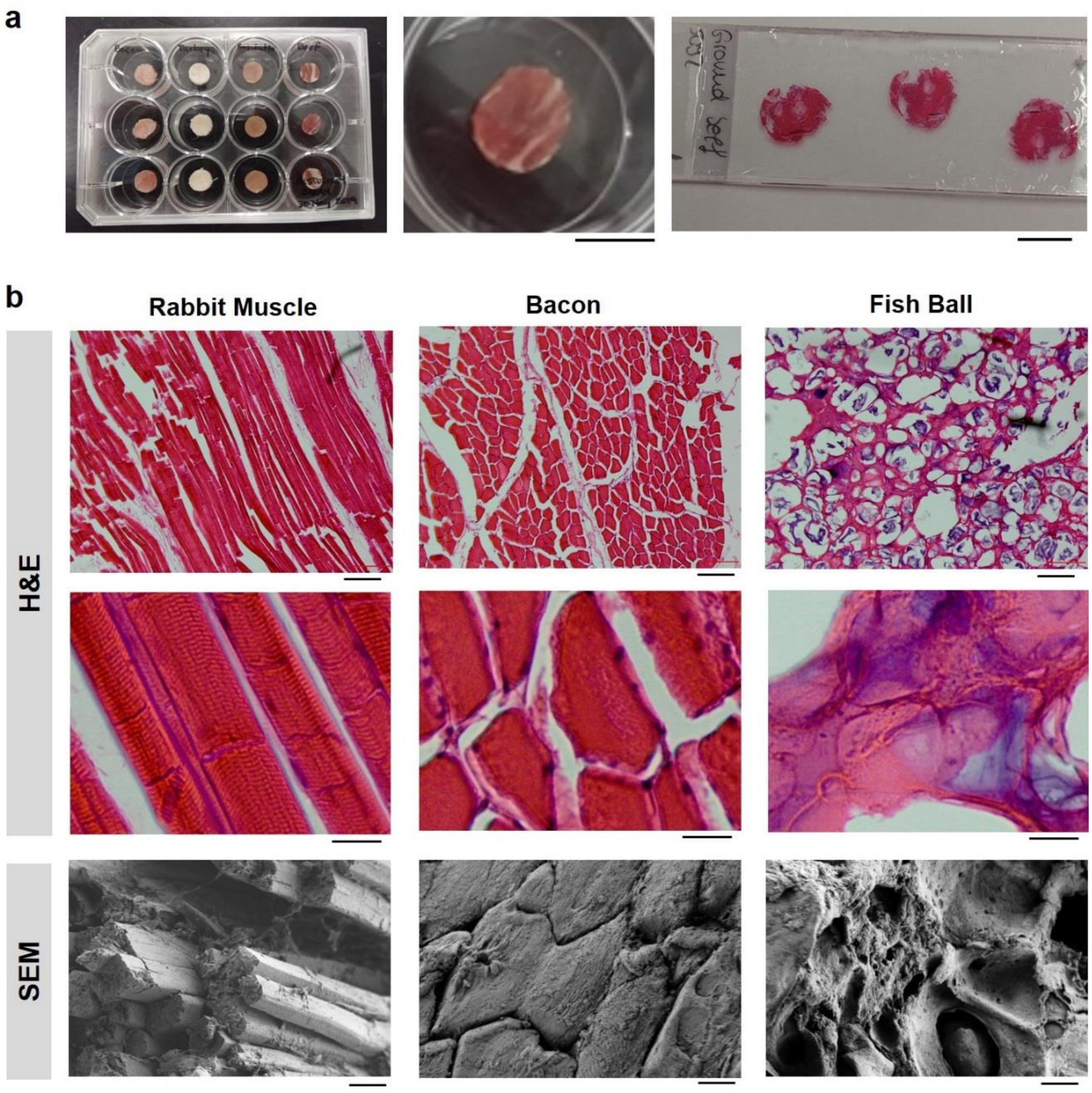

Figure S11 Sample preparation and representative microstructural comparisons of food products. a) Plated samples prepared by 1 cm biopsy punch of several meat products (including bacon, turkey breast, prosciutto, beef tenderloin, rabbit muscle, and ground beef). b) Hematoxylin and eosin (H&E) stains (top two rows) and scanning electron microscopy (SEM, bottom rows) of rabbit skeletal muscle (freshly isolated gracilis muscle from hind limb), bacon, and a processed 'fish ball' product. Scale bars are 200 μm (top row), 50 μm (middle row), and 20 μm (bottom row).

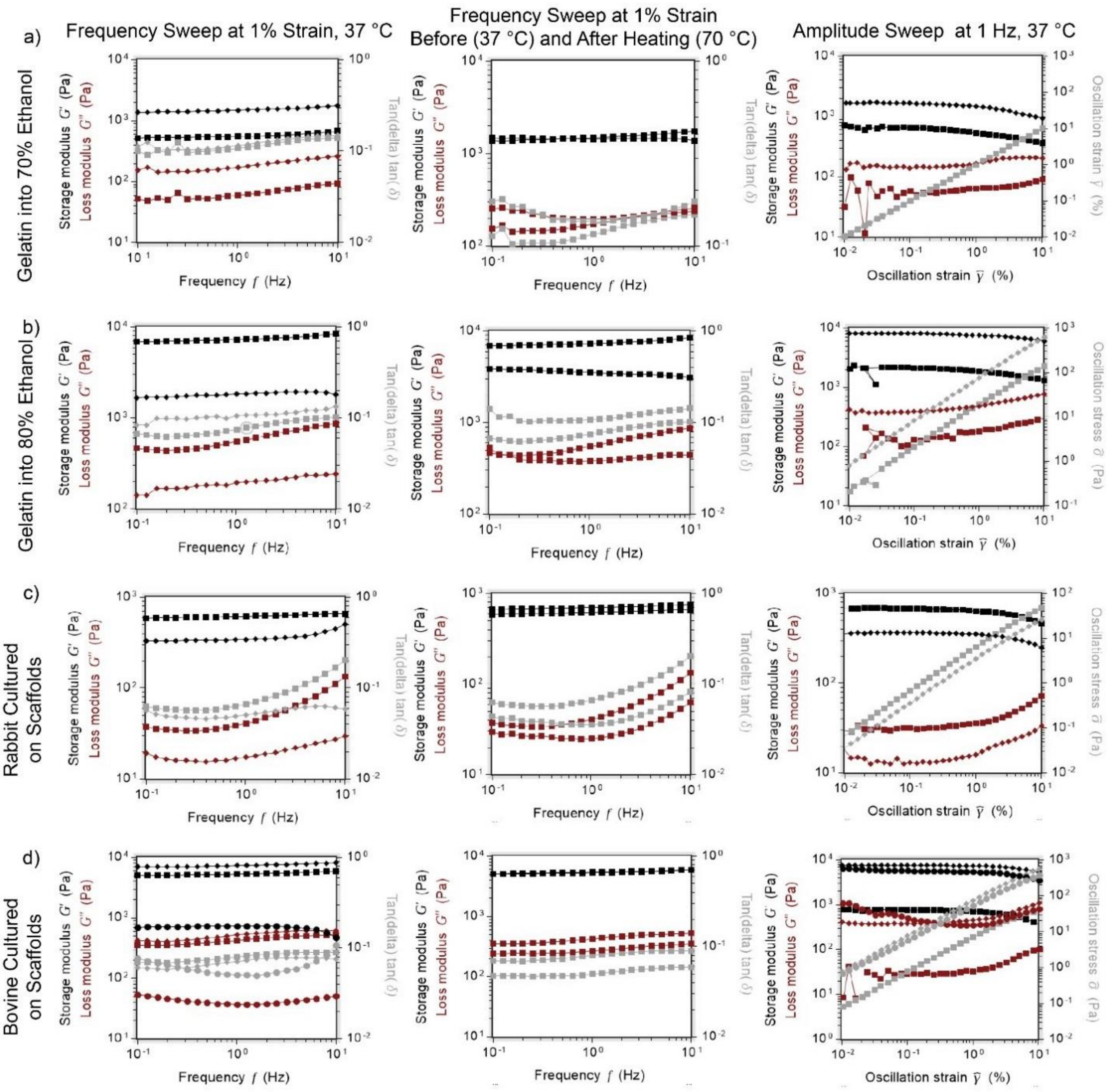

Figure S12 Rheological Mapping of Gelatin Scaffolds and Tissue Engineered Substrates Oscillatory mapping of scaffolds manufactured by spinning gelatin into a) 70% ethanol and b) 80% ethanol in addition to oscillatory mapping of c) rabbit cells seeded onto the scaffolds and d) bovine cells seeded onto the scaffolds. Frequency (at constant 1% strain) and Amplitude sweeps (at constant 1 Hz) occurred at 37°C. Frequency sweeps before heating occurred at 37°C and 70°C after heating (at constant 1% strain). Each curve of the same measurement repeats independent replicates except for the frequency sweep before and after heating where each curve is for either 37°C or 70°C.

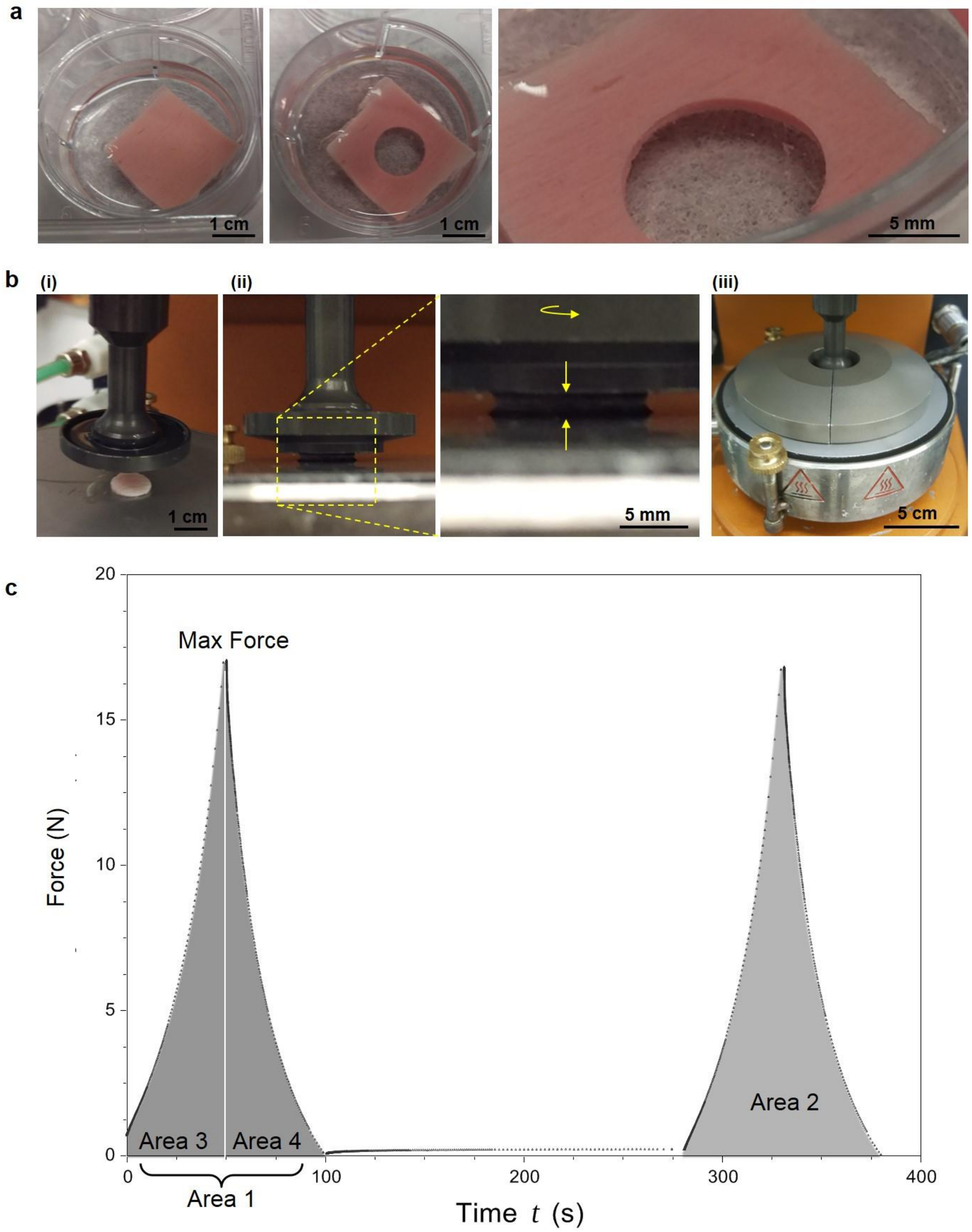

Figure S13 Sample preparation and representative force curves obtained by texture profile analysis (TPA). a) Tissue cultured sample with 1 cm diameter hole removed by biopsy punch. b) Cylindrical sample placed between plates in a rheometry system (i) was subjected to compression and shear tests (ii) within an evaporation trap that prevented solvent evaporation (iii). c) Force curves obtained by running two sequential compressions on a tissue cultured sample for TPA. Maximum force during compressions and areas under the curves are used to estimate TPA parameters.

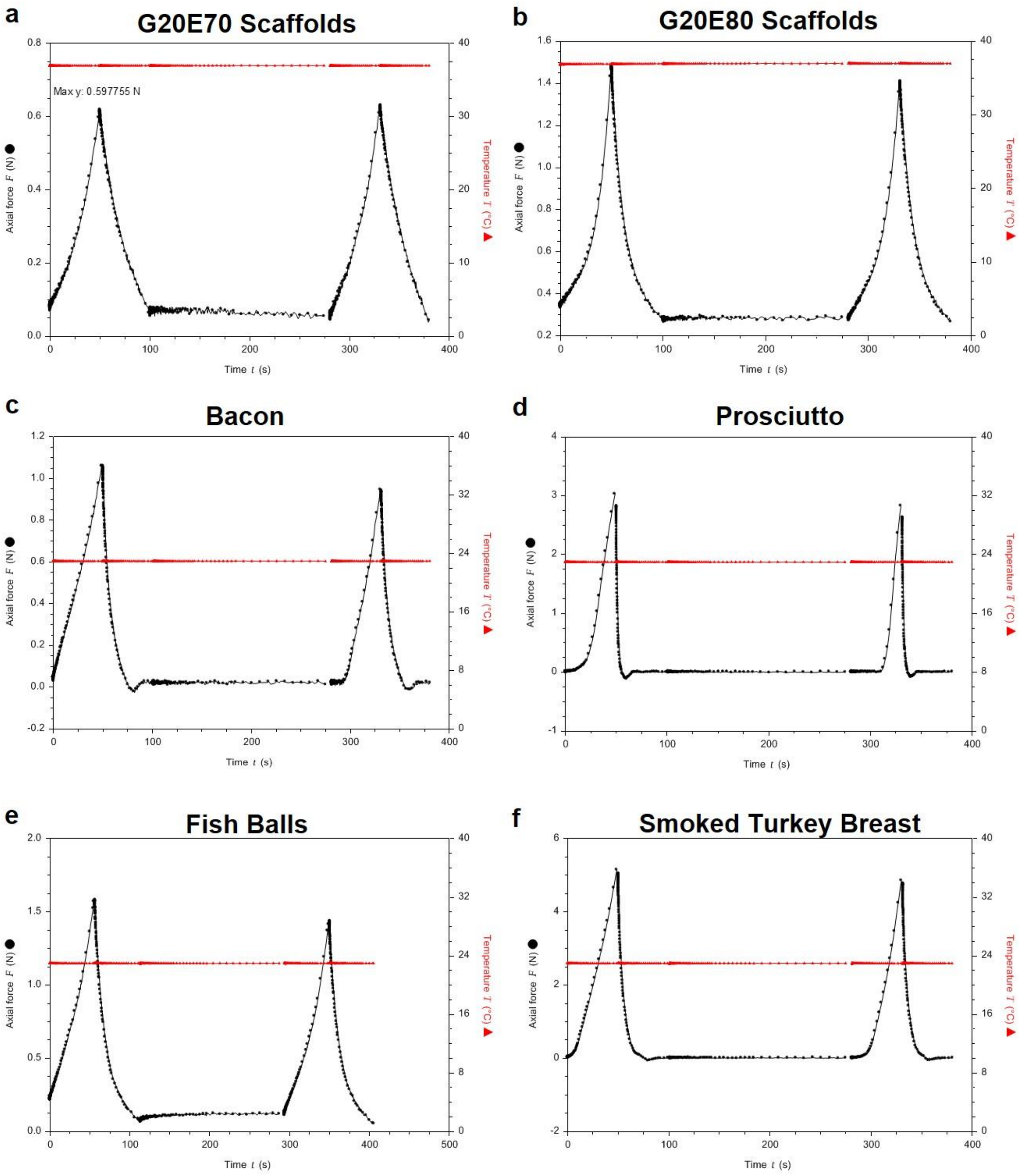

Figure S14 **T**exture profile analysis (TPA) dual compression curves for fibrous gelatin scaffolds (a, b) and selected food products (c-f). a,b) Fibrous gelatin scaffolds obtained by dissolving 20% w/w gelatin in water and spinning fibers into precipitation baths composed of either 70:30 Ethanol:water (a, G20E70) or 80:20 Ethanol:water (b, G20E80). c-f) TPA curves obtained by compression of bacon (c), prosciutto (d), processed fish balls (e), and smoked turkey breast (f).

SUPPLEMENTARY TABLES

Supplementary Table 1. Residual mTG detected by ELISA

Sample	mTG % (w/w) ± s.e.m
G20E80	2.80E-03 ±0.000268 (N=4)
G20E100	1.55E-03 ±6.83E-05 (N=2)
RbSkMC	7.60E-06 ±1.05E-06 (N=4)
BAOSMC	1.69E-05 ±3.33E-07 (N=2)
Prosciutto	4.40E-07 (N=1)
Beef Ball	1.71E-07 (N=1)
Fish Ball	5.16E-05 (N=1)
*Detection limit: 5.0E-07; N: Sample Number<br>Legend: G20E80; gelatin fibers from 20% gelatin spun into 80:20 ethanol:water, G20E100; gelatin fibers from 20% gelatin spun into 100% ethanol, RbSkMC; rabbit skeletal muscle myoblast cultured tissues, BAOSMC; bovine aortic smooth muscle tissues	

Supplementary Table 2. Residual mTG before and after culture

**Sample**	**mTG % (w/w)**
Scaffolds before cell culture	2.38E-03 ±0.000301 (N=6)
Tissues after cell culture	1.07E-05 ± 1.93E-06 (N=6)
Elisa detection limit: 1.0E-07; N: Sample Number	

Supplementary Table 3. Young's modulus and texture profile analysis (TPA) parameters from uncooked gelatin fiber scaffolds, cultured tissues, and food products

Sample	Sample size (n)	Young's Modulus (kPa)	Hardness (N)	Resilience (%)
G20E70	2	122.3 ± 45.4	1.4 ± 0.6	94.8 ± 1.3
G20E80	1	89.4	1.43	88.9
G20E70 RbSkMC D21	1	142.5	0.69	82.3
G20E80 BAOSMC D21	1	389.4	9.7	87.6
Beef Tenderloin	3	227.6 ± 82.9	2.4 ± 0.8	8.2 ± 1.3
Ground Beef	2	207.4 ± 125.3	3.3 ± 2.0	11.4 ± 1.0
Rabbit Skeletal Muscle	1	379.7	4.2	13.6
Processed Fish Ball	3	150.4 ± 53.0	1.5 ± 0.2	48.3 ± 3.1
Bacon	3	59.2 ± 23.6	0.7 ± 0.2	34.6 ± 3.7
Turkey	2	169.4 ± 95.1	5.3 ± 0.2	23.6 ± 0.4
Prosciutto	3	82.2 ± 7.6	3.4 ± 1.6	8.3 ± 2.3
*All data are mean ± standard error of the mean. For each sample compression, 25% peak strain was attained by a 50 s linear ramp followed by a 50 s withdrawal and a 180 s inter-compression period.<br>Legend: G20E70; gelatin fibers from 20% gelatin spun into 70:30 ethanol:water, G20E80; gelatin fibers from 20% gelatin spun into 80:20 ethanol:water, 'G20E70 RbSkMC D21'; rabbit skeletal muscle myoblasts cultured in G20E70 for 21 days, 'G20E80 BAOSMC D21'; bovine aortic smooth muscle cultured in G20E80 scaffolds for 21 days.				
